# Ethnobotanical survey and quantitative assessment of medicinal plants in landlocked communities of San Fernando, La Union, Philippines

**DOI:** 10.3389/fphar.2025.1670496

**Published:** 2025-11-13

**Authors:** Referenda Joanna V. Flores, Cecilia S. Cordero, Grecebio Jonathan D. Alejandro

**Affiliations:** 1 The Graduate School, University of Santo Tomas, Manila, Philippines; 2 Senior High School, University of Santo Tomas, Manila, Philippines; 3 Division of Biological Sciences, College of Arts and Sciences, University of the Philippines Visayas, Iloilo, Philippines; 4 College of Science, University of Santo Tomas, Manila, Philippines

**Keywords:** ethnobotany, ethnobotanical indices, medicinal plants, san fernando, la union, traditional medicines

## Abstract

**Introduction:**

Knowledge of medicinal plants is vital for preserving biodiversity, cultural heritage, and community healthcare. In San Fernando, La Union, traditional healing practices remain largely undocumented despite generational reliance on medicinal plants.

**Methods:**

This study documented medicinal plants used in three landlocked barangays (Bacsil, Saoay, and Abut). Modified semi-structured interviews were conducted in October 2024 with 252 informants aged 20–88 years (10.6% of the total population), reaching data saturation at interview 215. Ethnobotanical indices, including Use Report (UR), Use Value (UV), Fidelity Level (FL), and Informant Consensus Factor (ICF), were calculated following established protocols to evaluate the cultural significance of plant species.

**Results:**

A total of 93 plant species from 86 genera and 45 families were identified, treating 93 medical conditions across 17 disease categories. Fabaceae was the most represented family (11 species), followed by Lamiaceae and Poaceae (7 species each). Leaves were the most used plant part (62.3%), primarily prepared as decoctions (71.8%) for oral administration (68.4%). *Vitex arvensis* Gentallan, Sengun and M.B. Bartolome ranked highest across all ethnobotanical indices (UV = 1.54, RFC = 0.71, RI = 1.00), indicating broad medicinal applications and strong cultural recognition. Statistical analyses revealed significant geographic variation in knowledge (Kruskal-Wallis H = 45.23, *p* < .001): Barangay Saoay informants cited fewer species (5.2 ± 2.1) than Barangay Abut (8.4 ± 3.2; Mann-Whitney U, *p* < .001) and Bacsil (8.1 ± 2.9; Mann-Whitney U, *p* < .001). No significant differences were observed across gender (Mann-Whitney U, *p* = .909), civil status (Mann-Whitney U, *p* =.641), occupation (Kruskal-Wallis H, *p* = .564), education (Kruskal-Wallis H, *p* = .378), or age (Kruskal-Wallis H, *p* = .173).

**Discussion:**

This research documents rich ethnobotanical knowledge in landlocked communities and demonstrates how geographic isolation influences knowledge distribution, providing quantitative foundations for conservation and future pharmacological investigations.

## Introduction

The Philippines holds an enormous amount of traditional plant knowledge that’s still largely unstudied. The country has over 13,500 plant species, with around 1,500 known for medicinal uses (Galvez-Tan and Sia, 2014). Traditional medicine remains important, especially in remote areas where people have limited access to hospitals and clinics. The WHO has noted that these practices remain deeply embedded in Philippine healthcare, particularly for marginalized communities (Rubio and Naive, 2018; Suba et al., 2019; Agapin, 2020). Ethnobotanical work across the Philippines shows how much medicinal plant knowledge varies from one location to another. Some communities use as few as 20 species, while others know over 140, depending on whether they’re indigenous or non-indigenous, their level of isolation, elevation, and the extent of modernization that has reached them. However, the research coverage is patchy. Some provinces have been studied extensively, while others, such as La Union, have barely been touched.

Across the country, medicinal plant knowledge varies notably among indigenous and local communities. In Mindanao, most ethnobotanical research is focused on indigenous groups such as the Subanen, who were recorded using 89–113 species (Pizon et al., 2016; Alduhisa and Demayo, 2019); the Manobo, 40–122 species (Paraguison et al., 2020); and the Mamanwa and Talaandig, 48–97 species (Nuneza et al., 2021; Naive et al., 2021). Non-indigenous groups were documented using 65–70 species (Gruyal et al., 2014; Docot et al., 2022). In the Visayas, the Ati utilize 106–142 species (Cordero et al., 2020; Cordero and Alejandro, 2021), and the Panay Bukidnon 127 species (Cordero et al., 2022). Non-indigenous communities have documented 20–111 species, with rural areas showing more diversity than urban ones (Calangi et al., 2019; Cordero et al., 2023; 2025; Tiquio et al., 2024). In Luzon, the Kalanguya use 125 species (Balangcod and Balangcod, 2011), Batan Islanders use 112 (Abe and Ohtani, 2013), and Ayta communities use 54–118 species (Obico and Ragrario, 2014; Tantengco et al., 2018). Mixed communities have been documented using 74–106 species (Balangcod and Balangcod, 2015; Belgica et al., 2021; Caunca and Balinado, 2021).

Despite the documented importance of ethnobotanical knowledge across Southeast Asia (Monib, 2024), significant methodological and geographical gaps remain. Recent studies in Thailand (Phumthum et al., 2020), Vietnam (Nguyen and Nguyen, 2024), and Indonesia (Widodo et al., 2023) have demonstrated that elevation, market access, and healthcare infrastructure influence medicinal plant diversity and knowledge retention. However, the Philippines, particularly Luzon’s landlocked agricultural communities, remain critically understudied. Given global trends toward biocultural homogenization and the decline of traditional ecological knowledge among younger generations (Reyes-García et al., 2013; Aswani et al., 2018), documenting these systems is increasingly urgent.

La Union Province exemplifies this critical research gap. Of its 19 municipalities and one city, only the municipality of Santol has been studied, documenting 109 medicinal plant species (Ducusin, 2017). The rest of the province remains unexplored despite its ecological gradients from coastal to mountainous zones. Communities such as San Fernando’s landlocked barangays suggest the persistence of rich yet undocumented ethnobotanical traditions. Limited healthcare access (8.3–15.8 km from nearest hospitals), elevation-influenced plant diversity (38.6–166.1 m.a.s.l.), and shared Ilocano cultural traditions indicate structured yet insufficiently documented knowledge systems. The convergence of these factors creates an ideal natural laboratory for examining how environmental and sociodemographic variables shape ethnobotanical knowledge in semi-urban agricultural contexts.

However, this traditional knowledge is increasingly at risk of erosion due to urbanization, land conversion, and changing healthcare preferences. In remote mountainous barangays, the smallest administrative and political units in the Philippines (Porio and Roque-Sarmiento, 2019), restricted access to healthcare and economic constraints compel residents to rely on medicinal plants as practical alternatives to modern medicine. Documenting and preserving this knowledge is therefore essential to protect cultural heritage, support sustainable resource management, and provide a foundation for future research on medicinal plant use.

Therefore, this study aims to conduct ethnopharmacological documentation of the medicinal plant knowledge of locals in the three chosen barangays in San Fernando City, La Union. It specifically aims to: i. identify the medicinal plant species used and the disease they can treat, ii. describe the methods of preparation and administration of these medicinal plants, iii. evaluate the cultural significance of documented medicinal plants using quantitative ethnobotanical indices, and iv. assess variations in medicinal plant knowledge across sociodemographic factors such as age, gender, civil status, occupation, and education. These objectives address a central research question: What medicinal plants do locals use in three landlocked barangays of San Fernando, La Union, and what is their cultural and ethnopharmacological significance?

## Materials and methods

Our methodological approach was designed to address each component of the research question. Semi-structured interviews and plant collection enabled documentation of species and their medicinal uses. Detailed recording of plant parts, preparation techniques, and administration methods captured traditional practices. Ethnobotanical indices (UV, UR, RFC, RI, FL, ICF) quantified the cultural significance, while statistical analyses, including Shapiro-Wilk tests for normality and Mann-Whitney U and Kruskal-Wallis H tests for group comparisons, were performed.

### Study area

The study was conducted in the City of San Fernando, province of La Union. This location was selected with the help of the Department of Environment and Natural Resources (DENR) Region 1, which recognized the importance of ethnobotanical research in the area. The ecological diversity of the province, transitioning from sea level to montane environments, suggests the presence of varied plant assemblages that may support distinct local knowledge systems. However, documentation of these systems remains scarce amid rapid socio-economic and cultural changes in the region. Three barangays were selected for ethnobotanical study: Barangay Abut, Barangay Bacsil, and Barangay Saoay. This is to ensure a more focused and in-depth documentation, as well as practicality in terms of time, resources, and logistics. These barangays are located in landlocked mountainous areas and are away from the city center, where locals still rely on available plant resources for primary healthcare. They were identified with the help of the city’s Department of Public Safety (DPS), ensuring that the communities are accessible, safe to travel to, and that medicinal plant use is still practiced. Preliminary consultations with the DENR Region 1 and local health officials further revealed that while traditional medicinal plant knowledge persists, it is threatened by urbanization and shifting healthcare practices across generations.

San Fernando City, La Union (16°37′N, 120°19′E), is the only component city in the province and serves as both the provincial capital and the regional center of the Ilocos Region. It covers a total of 102.72 km^2^ and is bordered by the Municipality of San Juan to the north, Bauang to the south, Bagulin and Naguilian to the east, and the West Philippine Sea to the west. The city experiences a tropical and maritime climate characterized by relatively high temperatures (25.50 °C and 28.30 °C), high humidity (71%–85%), and abundant rainfall (965–4,064 mm annually). Based on the distribution of rainfall, the dry season occurs from November to April, and the wet or rainy season from May to October (PAGASA, 2025). San Fernando City is primarily agricultural, where locals cultivate root crops, vegetables, legumes, fruits, rice, and corn. Livestock production is also important to the locals, which includes chickens, hogs, goats, cattle, and carabaos (GOVPH, 2025).

**FIGURE 1:**
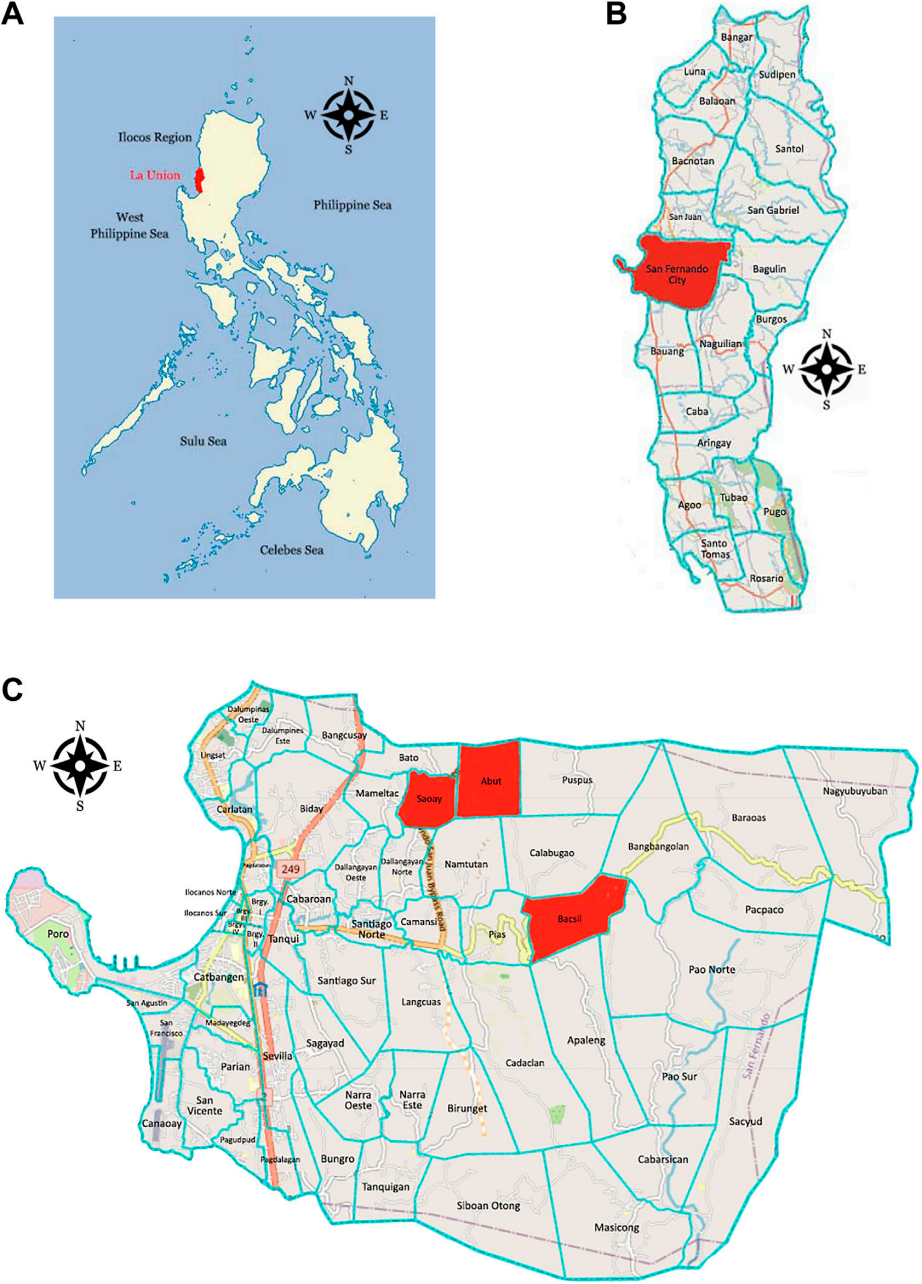
Location of the study site: **(A)** The Province of La Union in the Philippines (PhilAtlas (n.d.), 2025). **(B)** The City of San Fernando in La Union. **(C)** Barangay Abut, Bacsil, and Saoay, City of San Fernando, La Union (DOST-PHIVOLCS. (n.d.)).

There are 59 barangays of San Fernando City: 10 are coastal barangays directly facing the West Philippine Sea, 27 are near-coastal barangays located close to the shoreline but not directly on it, and 22 are landlocked barangays situated inland, mostly within the eastern mountainous regions. From the 22 landlocked barangays, three were selected as study sites: Barangay Abut (16°38′N, 120°21′E; 71.6 m.a.s.l.), Barangay Bacsil (16°37′N, 120°22′E; 166.1 m.a.s.l.), and Barangay Saoay (16°38′N, 120°20′E; 38.6 m.a.s.l.). These sites share cultural and linguistic characteristics rooted in Ilocano traditions but differ in key ecological aspects, particularly elevation, which likely influences vegetation composition and plant availability. These barangays are geographically isolated from the city center, with Barangay Abut located 11.1 km, Barangay Bacsil 15.8 km, and Barangay Saoay 8.3 km from major hospitals. This distance creates continued dependence on locally available resources for primary healthcare. The DPS confirmed that these barangays were accessible and safe for travel, and that local residents were cooperative and supportive of the research activities. The locations of the study sites are shown in Figure 1.

As of October 2024, Barangay Abut had a population of 642 individuals, Barangay Bacsil had 722 residents, and Barangay Saoay had 1,022 residents. These figures are based on the most recent population data provided by the barangay officials, as the official results of the 2024 national census have not been officially released yet.

A permit to conduct this research was granted by the Office of the Mayor. A Wildlife Gratuitous Permit (No. 2024-008) was also issued by the Department of Environment and Natural Resources (DENR) Region 1 for the collection of voucher specimens.

### Sampling and interview

A modified semi-structured questionnaire, adapted from Longuefosse and Nossin (1996) and supplemented with open-ended questions, was used to conduct interviews with informants. The questionnaire was prepared in the Ilocano dialect, with English translations to ensure clear communication and understanding. To ensure ethical compliance, the research protocol was evaluated and approved by the Ethics Review Committee of the University of Santo Tomas Graduate School (REC Code: 2024-052). All informants provided written informed consent prior to participation. For informants with limited literacy (n = 3), verbal consent was obtained in the presence of a barangay official witness, with thumbprint documentation as approved by the ethics committee. Consent forms, available in both Ilocano and English, explicitly outlined: 1. study objectives and procedures, 2. voluntary nature of participation and right to withdraw, 3. data confidentiality and anonymization protocols, 4. absence of direct benefits or compensation, and 5. researcher contact information for queries or concerns.

To ensure participant anonymity, all identifying information was removed during the data encoding process. Informants were assigned unique alphanumeric codes (e.g., AB-001 for Barangay Abut, informant 1), and only aggregate demographic data are reported. Physical questionnaires are stored in a locked cabinet accessible only to the principal investigator, while electronic data are password-protected and backed up on encrypted storage. Data will be retained for 5 years after publication, as per institutional policy, after which both physical and electronic records will be permanently destroyed.

The questionnaire is composed of three parts. The first part includes the personal profiles of the respondents. The second part includes the health problems experienced by the respondents and the medicinal plants they used. It contains a set of inquiries regarding the health issue that the respondents faced, the steps they took to tackle the problem, the local names of the medicinal plants they used, the specific parts of the plant that were used, the different forms, methods, and frequency of administration, how they obtained or passed on this knowledge, and the possibility of side effects or adverse reactions. Lastly, the third part is the tabulated form of the Part II questions. It is designed for respondents who are familiar with multiple health problems and medicinal plants used to treat various ailments.

During the interview, the informants were asked to describe their current ailments as well as any past illnesses they had experienced. Follow-up questions were included to document their personal experiences, such as what they felt when they had an ailment, what they thought could have caused it, and if they consulted a medical doctor about it. If they did not seek medical advice, they were asked to explain why. For example, what did they feel when they had a stomachache? What could have been the cause? Did they consult a medical doctor regarding their condition? If not, why? This will serve as validation that their claim about their ailment is not based on personal perception or self-diagnosis but rather supported by a medical consultation. Field notes from site visits were also taken to further document additional details needed in the study. These notes were taken not only during the interviews but also during any subsequent group discussions.

The survey and interviews were conducted from October 7 to 11, 2024, using purposive and snowball sampling techniques. Purposive sampling relies on the researcher’s judgment in selecting participants who possess relevant knowledge or expertise (Elfil and Negida, 2017; Berndt, 2020), ensuring the collection of rich and meaningful data for ethnobotanical analysis (Tongco, 2007; Suter, 2011). Snowball sampling involves identifying and recruiting additional participants through referrals from initial informants (Mincheva et al., 2022), which is particularly effective when studying communities with a limited number of knowledgeable individuals (Lewis-Beck et al., 2004).

The study focused on documenting household-level medicinal plant knowledge among the general population rather than traditional healers. Informants were primarily community members, including farmers, housewives, barangay officials, and other residents who possess practical knowledge passed down through generations and apply it in their daily lives. This approach aligns with community-based ethnobotanical practices, which are consistent with the study’s objective.

Key informants included barangay officials and barangay health workers, not only participated as respondents but also assisted in identifying other informants and facilitating the house-to-house survey and interview. With proper briefing and instructions, approximately 5-6 health workers and 2-3 barangay officials helped coordinate the data collection. Each informant completed the survey individually, although interviews were sometimes conducted in small groups, especially among members of the same household. This inclusive approach enables efficient community engagement and representation of diverse sectors, each contributing unique perspectives on the use of medicinal plants. The sample size was determined, data saturation was monitored, and informant reliability was assessed throughout the study. Detailed computations and discussions are provided in Supplementary Material 1.

All participants signed informed consent forms and completed surveys that collected data on their personal background, health conditions, and the medicinal plants they used. The survey also documented information on plant parts used, preparation methods, modes of administration, and any observed side effects. Quantitative data were analyzed using Microsoft Excel (Microsoft 365, Version 2501).

### Plant collection, identification, and voucher specimen preparation

Plant samples were collected with the assistance of informants, who guided researchers to the locations of medicinal species. The collection was not restricted to the interview period but was extended until May 2025 to ensure that more plants were in their flowering condition. This will help facilitate the accurate identification of the documented medicinal plant species. Photographs of the plants were also taken for documentation and confirmation. Key plant features, including leaves, flowers, fruits, seeds, bark, and roots, were collected for each species. Information such as the collection date, habitat, and plant habit was recorded. For each species, 2–3 branches (preferably with reproductive parts) were collected. The specimens were initially placed between sheets of newspaper inside polyethylene bags, with denatured alcohol added to preserve them during transport.

At the laboratory, specimens were transferred to fresh newspapers, pressed in a plant metal presser, and oven-dried for 48–72 h. After drying, specimens were mounted and labeled on 45.72 cm × 30.48 cm herbarium sheets (Bristol board). All mounted specimens were deposited at the University of Santo Tomas Herbarium (USTH). Specimen identification employed a multi-tiered validation approach. Preliminary identification and classification of plant species were conducted using multiple online taxonomic databases: World Flora Online (WFO, 2025) for species identification, StuartXchange (Stuart, 2025) and Plants of the World Online (POWO, 2025) for growth form classification; Co’s Digital Flora of the Philippines (Pelser et al., 2011 onwards) for endemicity or distribution, and the International Union for Conservation of Nature (IUCN) Red List of Threatened Species (IUCN, 2024) for conservation status. Specimens were then verified independently by two taxonomic experts: Niña Kathryn G. Alfeche, curator of the UST Herbarium, and Grecebio Jonathan D. Alejandro, plant taxonomist. In cases of taxonomic uncertainty (n = 7 species), specimens were cross-referenced with authenticated herbarium vouchers at the Philippine National Herbarium (PNH) and validated through consultation with regional specialists from the Department of Environment and Natural Resources (DENR) Region 1. This multi-expert verification approach reduces identification bias inherent in single-expert determinations (Prance et al., 2007). Additionally, high-resolution photographs were taken for all specimens to facilitate future re-identification if necessary.

### Quantitative data analysis of medicinal plants

#### Use Value (UV)

The UV was used to evaluate a plant’s relative importance within a community. It is calculated as UV = U/N, where U is the number of use report (UR) for a species and N is the total number of informants (Polat et al., 2015). A high UV suggests frequent and diverse use of the plant. UR refers to the number of times a plant is cited or mentioned for treating a particular condition. A single UR was recorded each time a plant was cited for a disease or purpose within a category. Multiple URs were noted if multiple informants mentioned the same plant for the same use.

#### Informant consensus factor (ICF)

The Informant Consensus Factor (ICF) measures the degree of agreement among informants on the use of plants for specific disease categories. It is computed using ICF = (N–N)/(N–1), where N is the number of URs in each category, and N is the number of species cited for that category. An ICF of 1.00 indicates complete consensus; values closer to 0 indicate minimal or no consensus (Teklehaymanot and Giday, 2007; Aswathi and Abdussalam, 2021; Dangwal and Lal, 2024).

Unit of analysis and data structure, data validation and statistical assumptions, and disease categories and computation of other indices are shown in Supplementary Material 2.

### Statistical analyses

As a supportive tool to strengthen data interpretation, statistical analyses were employed. All responses were encoded into a database and checked for consistency and completeness prior to analysis. The number of medicinal plants cited by each informant was quantified and used for univariate and nonparametric tests. A binary matrix was also generated for the 93 medicinal plant species, where 1 indicated citation and 0 indicated non-citation, to enable multivariate analysis. All statistical analyses were conducted using IBM SPSS Statistics, version 31.0.0.0 (117). To assess if the distribution of medicinal plant knowledge among informants is normal, the Shapiro-Wilk test for normality was employed. The Mann-Whitney U test was applied to evaluate differences in knowledge between two independent groups, such as gender and civil status. For comparisons of knowledge across three or more groups, specifically barangay, occupation, educational attainment, and age group, the Kruskal-Wallis H test was employed, followed by a *post hoc* pairwise Mann-Whitney U test to identify specific sources of variation.

## Results

### Demographic profile of informants

More than 10% of the total population from each selected barangay participated in the study (Table 1). A total of 252 informants were interviewed, comprising 44% males and 56% females, with ages ranging from 20 to 88 years. Most of the informants are married (76%), while some are single (24%). Other demographic profiles of the informants are shown in Table 1.

**TABLE 1:** Demographic profile of informants.

Social	Variable	Number of informants			Total	Percentage
Category		Abut	Bacsil	Saoay		
Gender	Male	31	31	48	110	43.65
	Female	36	48	58	142	56.35
Civil Status	Single	18	12	31	61	24.21
	Married	49	67	75	191	75.79
Education	Elementary	14	13	18	45	17.86
	High school	29	41	60	130	51.59
	College/Vocational	22	25	27	74	29.36
	Did not attend school	2	1	0	3	1.19
Occupation	Employed	33	30	45	108	42.86
	Housewife	19	26	35	80	31.75
	Farmer	11	18	18	47	18.65
	Unemployed	4	5	8	17	6.74
Age	20–39	26	34	51	111	44.05
	40–59	32	25	27	84	33.33
	60–79	8	17	26	51	20.24
	≥80	1	3	2	6	2.38

All three barangays are Ilocano-speaking communities where most families farm vegetables, root crops, rice, and corn. Nearly all households have home gardens with medicinal plants growing alongside food crops. People share plant knowledge naturally during farm work, at community gatherings, and through family visits. Getting to hospitals is difficult. Saoay is 8.3 km away, Abut is 11.1 km, and Bacsil is 15.8 km from the nearest hospital. Some roads are unpaved, and public transport is limited. Many families cannot afford regular trips to town. This isolation, combined with the cost of medical care, explains why locals continue to rely heavily on plants they can grow or gather nearby.

### Medicinal plants used

**FIGURE 2:**
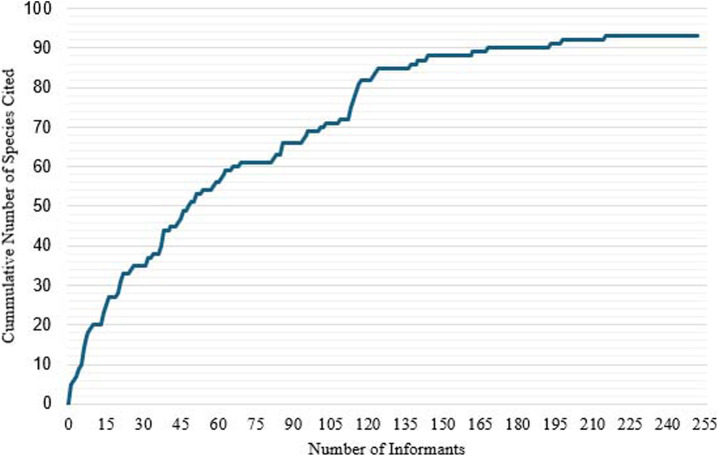
Data saturation curve of medicinal plant species reported.

A total of 93 plant species, representing 86 genera and 45 families, were documented as being used to treat approximately 93 medical conditions classified under 17 disease categories. No new species were cited after 215 interviews, which means data saturation was reached (Figure 2). Among the informants, 37% (n = 92) reported relying exclusively on medicinal plants to manage their health conditions. Meanwhile, 62% (n = 157) used medicinal plants in conjunction with consultations from medical doctors. Only 0.4% (n = 1) reported using medicinal plants under the guidance of a mystical healer or *albularyo* while also seeking medical advice from a physician. The majority of informants reported that their knowledge of medicinal plants came from their relatives (87.30%) and parents (81.35%). Some indicated that their knowledge was shared with them by the community (77.38%), while only a small portion mentioned social media (7.94%) and friends (0.40%) as sources of information.

**FIGURE 3:**
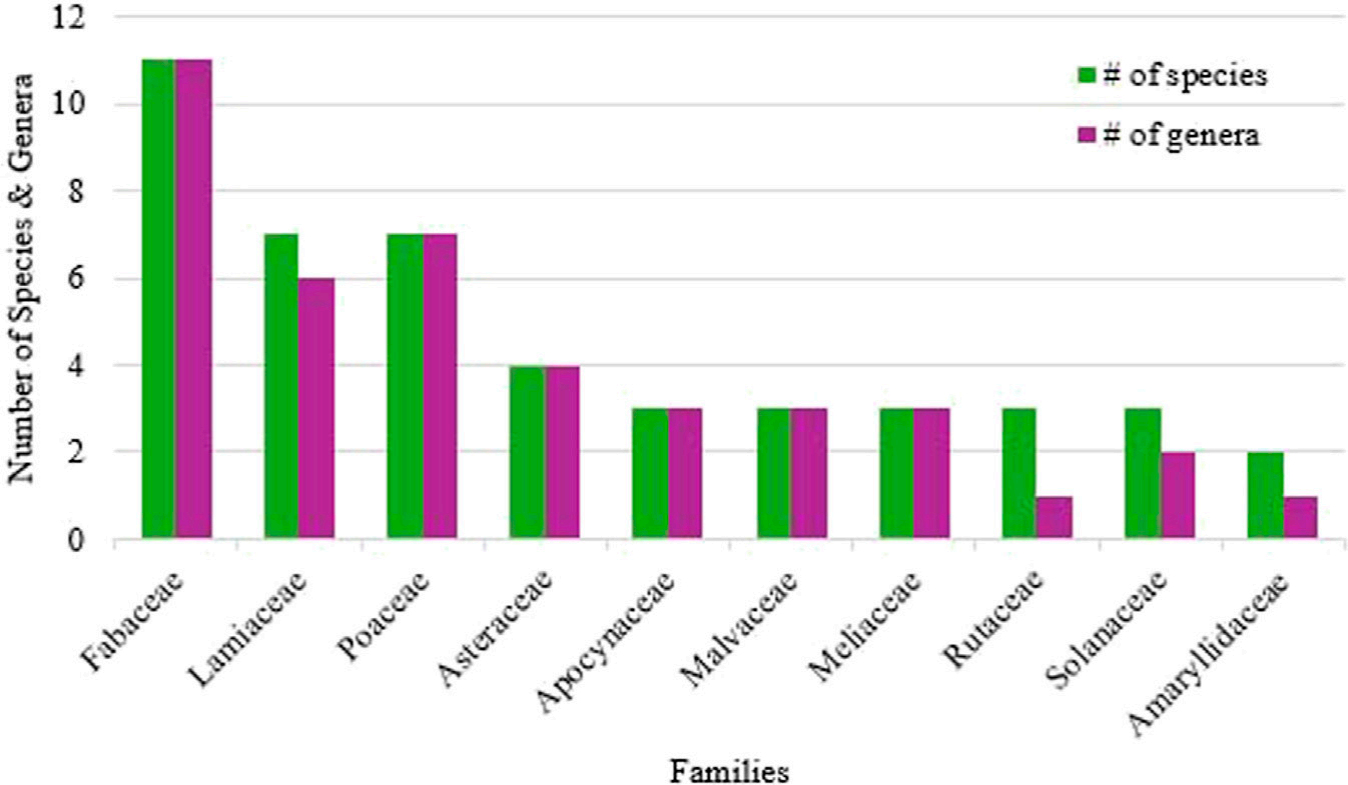
Plant families with highest species diversity and ethnobotanical importance. Values represent the number of species and genera per family, with corresponding use reports (URs). Fabaceae: 11 species, 11 genera, 47 total URs (mean = 4.3 ± 1.2 URs per species); Lamiaceae: 7 species, 6 genera, 603 total URs (mean = 86.1 ± 52.3 URs per species); Poaceae: 7 species, 7 genera, 126 total URs (mean = 18.0 ± 9.4 URs per species); Myrtaceae: 2 species, 2 genera, 91 total URs (mean = 45.5 ± 36.5 URs per species); Annonaceae: 2 species, 1 genus, 96 total URs (mean = 48.0 ± 33.0 URs per species); Solanaceae: 3 species, 2 genera, 17 total URs (mean = 5.7 ± 2.9 URs per species); Zingiberaceae: 2 species, 2 genera, 32 total URs (mean = 16.0 ± 4.0 URs per species); Rutaceae: 3 species, 1 genus, 17 total URs (mean = 5.7 ± 2.3 URs per species); Euphorbiaceae: 2 species, 2 genera, 41 total URs (mean = 20.5 ± 13.5 URs per species); Asteraceae: 3 species, 3 genera, 249 total URs (mean = 83.0 ± 65.2 URs per species). Error bars represent standard error of the mean. N = 252 informants.

In terms of plant families with the highest number of species, the Fabaceae family was the most represented, comprising 11 species across 11 genera, which are used to treat 27 medical conditions. This was followed by Lamiaceae, with seven species from six genera used for 27 medical conditions, and Poaceae, which included seven species from seven genera utilized in managing 26 medical conditions (Figure 3). The three most frequently cited medicinal plants were *Vitex arvensis* Gentallan, Sengun and M.B. Bartolome, with 178 citations (71%), *Coleus amboinicus* Lour. with 123 citations (49%), and *Blumea balsamifera* (L) DC. with 66 citations (26%). The documented medicinal plants include various growth forms, which include 41% (n = 38) trees, 37% (n = 35) herbs, 12% (n = 11) shrubs, and 10% (n = 9) vines.

Based on Pelser et al. (2011 onwards), the origin, adaptation, and establishment of documented medicinal plants were identified. Of the 93 species documented, 56% (43) were naturalized. Several of them are valued for their fruits (*Psidium guajava* L., *Mangifera indica* L.), some are legumes (*Psophocarpus tetragonolobus* (L.) DC., *Leucaena leucocephala* (Lam.) de Wit), and others are considered invasive (*Lantana camara* L., *Mimosa pudica* L.). About 34% (26) of the recorded species are native and have multiple uses: some are edible (*Ipomoea aquatica* Forssk., *Cocos nucifera* L.); others are sources of biofuel (*Jatropha curcas* L., *Gliricidia sepium* (Jacq.) Kunth.), while some provide quality timber (*Sandoricum koetjape*, *Alstonia scholaris* (L.) R.Br.). Two species (2%) were found to be endemic to the Philippines: *V. arvensis* Gentallan, Sengun and M.B. Bartolome, and *Areca catechu* L. *V. arvensis*, locally known as *lagundi*, is widely used to treat respiratory ailments such as cough, colds, and asthma. *A. catechu*, known as *bunga*, is a solitary-stemmed palm traditionally chewed by elders in rural communities. It is used to treat abdominal pains, intestinal worms, and to strengthen teeth. The cultivated species that are not naturalized constitute 20% (18) of the total and include herbaceous medicinal species (*C. amboinicus* Lour., *Cymbopogon citratus* (DC.) Stapf), staple crops (*Oryza sativa* L., *Zea mays* L.), fruit-bearing woody species (*Annona muricata* L., *Chrysophyllum cainito* L.), and plants used as natural pesticides (*Allium sativum* L., *C. citratus* (DC.) Stapf). Two species (2%) categorized as cultivated and not native (*Aloe vera* L., *Persea americana* Mill.) are rich in bioactive compounds and are commonly used in skincare and hair care products. Lastly, two (2%) cryptogenic species were recorded, which included edible species of uncertain origins, such as *Piper betle* L., typically chewed for its medicinal properties, and *Portulaca oleracea* L., consumed as a leafy vegetable.

According to the IUCN Red List of Threatened Species, 45% (42) of the documented species are not listed in the database. This includes a range of species commonly used for human consumption, such as staple crops (*O. sativa* L.*, C. nucifera* L.), fruits and vegetables (*Cucumis sativus* L., *Momordica charantia* L.), and herbs and spices (*A. sativum* L., *C. citratus* (DC.) Stapf). This group also includes ornamental plants primarily grown for their aesthetic value (*Hibiscus rosa-sinensis* L.*, Coleus scutellarioides* (L.) Benth.), as well as toxic species (*Abrus precatorius* L.*, Nicotiana tabacum* L.) and invasive species (*L. camara* L.*, L. leucocephala* (Lam.) de Wit). Approximately 47% (44) of the listed species are of Least Concern, which includes various fruit-bearing trees (*P. americana* Mill., *Tamarindus indica* L.), timber and industrial species (*A. scholaris* (L.) R.Br., *Samanea saman* (Jacq.) Merr.), a few toxic plants (*J. curcas* L.), and invasive or fast-growing weeds and trees (*Commelina benghalensis* L., *Eleusine indica* (L.) Gaertn.). Six (7%) species classified as Data Deficient are mainly culinary and aromatic plants such as spices (*Zingiber officinale* Roscoe, *Curcuma longa* L.), herbs (*Pandanus amaryllifolius* Roxb. ex Lindl., *Artemisia indica* Willd.), and fruit trees (*Carica papaya* L., *M. indica* L.). Only one (1%) species, *Swietenia macrophylla* King, commonly known as big-leaf mahogany, is listed as Endangered. Despite this classification, it is widely distributed in the Philippines, valued for its high-quality timber.

### Collection, preparation, and administration of medicinal plant parts

**FIGURE 4:**
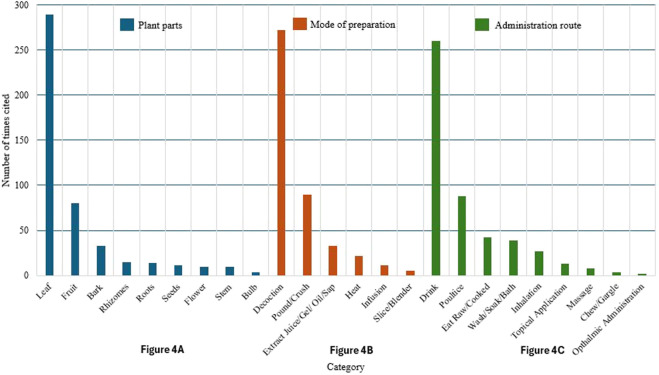
Distribution of plant parts used, preparation methods, and administration routes for medicinal plants (N = 252 informants, 93 species, total URs = 1,847). **(A)** Plant parts used: Leaves: 62.3% (n = 1,151 URs, mean = 4.6 ± 0.3 URs per informant); Roots: 8.2% (n = 151 URs, mean = 0.6 ± 0.1 URs per informant); Bark: 6.4% (n = 118 URs); Stems: 4.1% (n = 76 URs); Flowers: 5.8% (n = 107 URs); Fruits: 9.7% (n = 179 URs); Seeds: 2.3% (n = 42 URs); Rhizomes: 1.2% (n = 23 URs). **(B)** Mode of preparation: Decoction: 71.8% (n = 1,326 URs, mean = 5.3 ± 0.3 URs per informant); Infusion: 8.4% (n = 155 URs); Poultice: 12.6% (n = 233 URs); Direct consumption: 4.7% (n = 87 URs); Steam inhalation: 2.5% (n = 46 URs). **(C)** Route of administration: Oral: 68.4% (n = 1,263 URs, mean = 5.0 ± 0.3 URs per informant); Topical: 26.1% (n = 482 URs); Inhalation: 3.2% (n = 59 URs); Bathing/washing: 2.3% (n = 43 URs). Percentages calculated from total use reports. Error bars represent 95% confidence intervals.

Most of the medicinal plants used by the informants are locally sourced, with 47% collected directly within the community, and 42% cultivated in home gardens. A smaller portion, 8%, is collected from nearby forests or woodlands, while only 3% is purchased from other communities (3%). The most commonly used plant parts for medicinal purposes are leaves, roots, bark, stems, flowers, and fruits, with leaves being the most frequently utilized component due to their accessibility and ease of preparation. Moreover, preparation methods vary depending on the ailment being treated and the plant part used, with decoction (boiling plant parts in water) being the most common. Other methods include infusion, poultice application, direct consumption, chewing, steaming, and topical application of plant extracts. Prepared plant parts are usually administered through oral ingestion, topical application, inhalation, bathing, or fumigation. Oral administration is the most common method, especially for decoctions and infusions. This is followed by poultice, which is primarily applied for skin conditions, wounds, and muscle pains. Other routes, such as inhalation and fumigation, are practiced less frequently but are culturally significant in treating colds and spiritual ailments. A graph of the different plant parts used, mode of preparation, and administration route is shown in Figure 4.

### Disease categories treated with medicinal plants

Out of the 26 disease categories outlined in the International Classification of Diseases (ICD), 17 categories were represented in this study. An additional category, Other Diseases (DC00), was created to include ailments that do not fall within the ICD framework but are culturally recognized by the local community. This includes ailments such as *nakablaawan* and *pasma*. *Nakablaawan* is an Ilocano word that means “greeted.” It is locally perceived as a mystical illness that occurs when a person is “greeted” by spirits or a person with negative energy or with envy. The person “greeted” may feel symptoms such as sudden dizziness, nausea, headache, and discomfort. These symptoms are often sudden and unexplained, and treatment is usually sought from *albularyos*, who use rituals or herbal medicines. *Pasma* is a condition recognized in Filipino folk medicine, but it has no direct biomedical equivalent. It is described as a syndrome caused by abrupt exposure to extreme temperatures. One common example of this is when a person who is physically tired due to strenuous activity is subsequently exposed to cold water by taking a bath. Common reported symptoms include tingling sensations, numbness, limb tremors, and muscle stiffness. Treatment usually involves soaking the affected body parts in warm herbal decoctions, applying topical ointments, or massaging.

**FIGURE 5:**
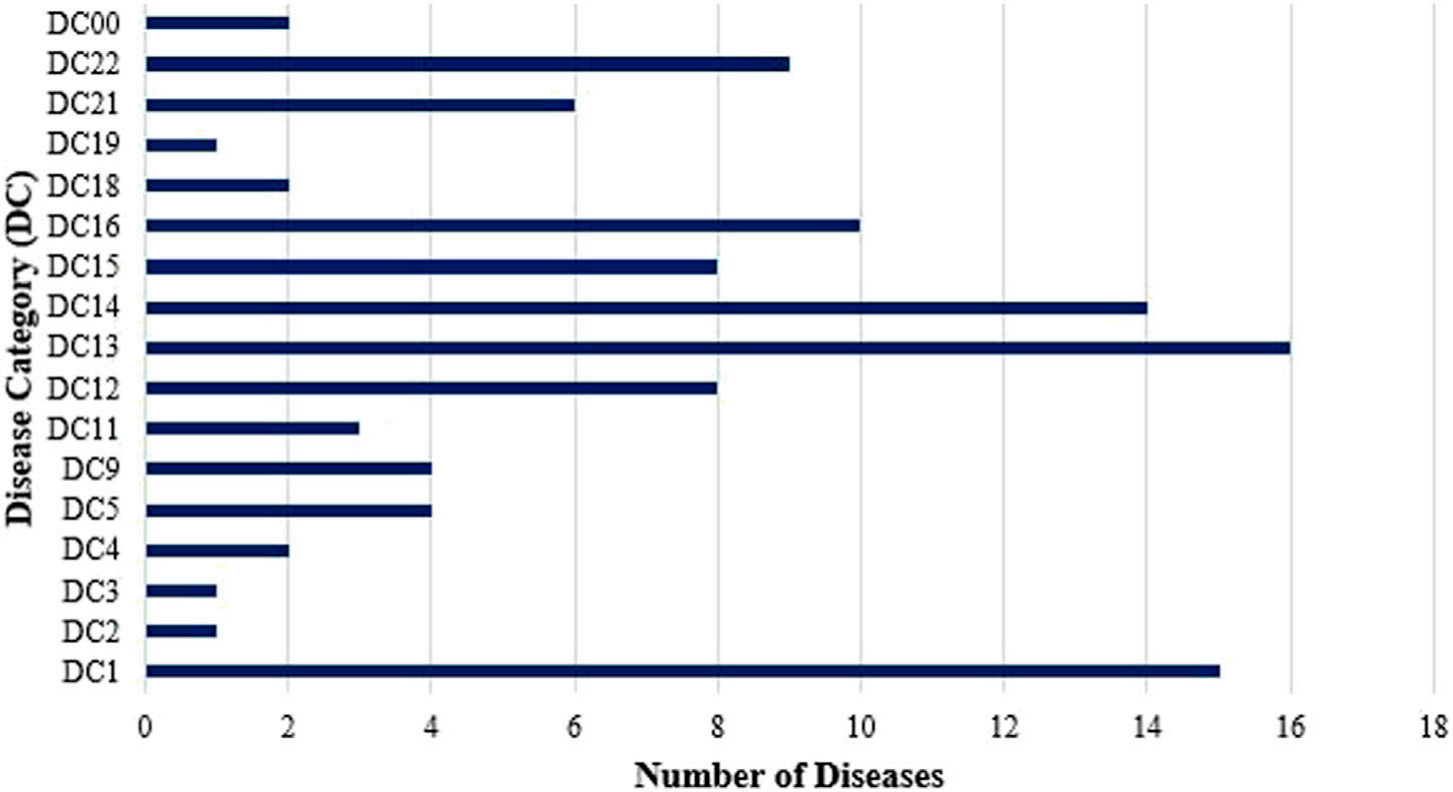
Distribution of medicinal plant use reports across disease categories based on ICD-11 classification (N = 252 informants, 93 species, 17 disease categories). Disease categories ranked by number of use reports (mean ± SE URs per category): DC13 (Digestive System): 141 URs (mean = 8.3 ± 2.8 species per category); DC1 (Infectious/Parasitic): 387 URs (mean = 11.1 ± 1.9 species); DC14 (Skin): 83 URs (mean = 5.9 ± 1.2 species); DC12 (Respiratory): 387 URs (mean = 6.5 ± 0.8 species); DC16 (Genitourinary): 223 URs (mean = 6.1 ± 1.1 species); DC11 (Circulatory): 125 URs (mean = 7.1 ± 1.4 species); DC22 (Injuries): 177 URs (mean = 5.1 ± 0.6 species); DC21 (Symptoms/Signs): 258 URs (mean = 5.2 ± 0.9 species); DC5 (Endocrine/Metabolic): 149 URs (mean = 7.8 ± 2.1 species); DC15 (Musculoskeletal): 55 URs (mean = 4.7 ± 1.3 species); DC18 (Pregnancy/Childbirth): 21 URs; Other categories: <20 URs each. Numbers in parentheses indicate total diseases documented within each category. Total diseases documented = 93 across all categories.

The three most frequently cited disease categories were the Diseases of the Digestive System (DC13), which include conditions such as constipation, diarrhea, dysentery, stomachache, and liver problems. This was followed by Infectious and Parasitic Diseases (DC1), encompassing ailments such as amoebiasis, dengue fever, malaria, measles, and tuberculosis. The third most reported category was Diseases of the Skin and Subcutaneous Tissue (DC14), which covers conditions such as abscesses, eczema, rashes, and ringworm. The other Disease Categories cited in the study are shown in Figure 5.

**FIGURE 6:**
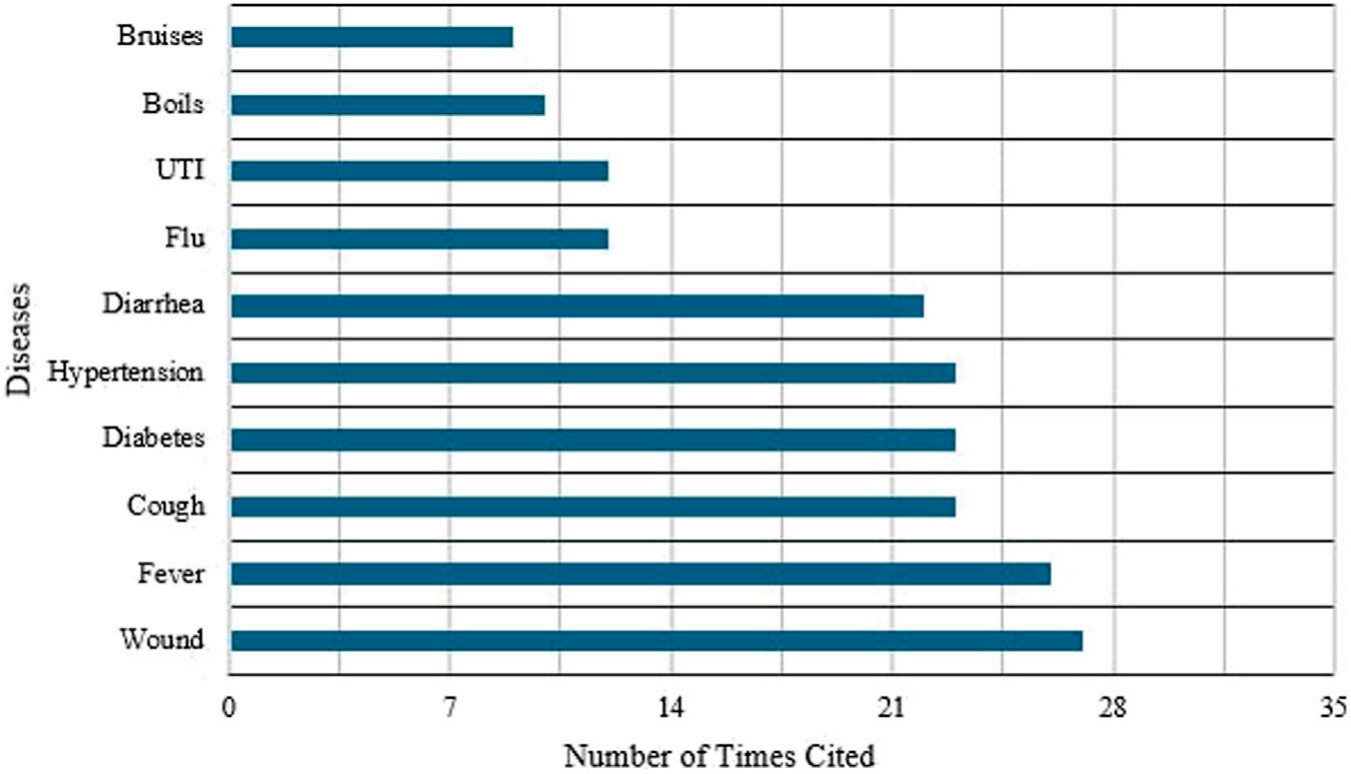
Top 10 most frequently cited medical conditions and number of plant species used for treatment (N = 252 informants, 93 total species). Conditions ranked by frequency of citation: Wounds: 78 URs, 35 species (mean = 2.2 ± 0.4 species per informant citing this condition, 22.3% of URs per species); Fever: 227 URs, 35 species (mean = 6.5 ± 0.8 URs per species); Cough: 352 URs, 26 species (mean = 13.5 ± 3.2 URs per species, highest mean utilization); Diabetes: 123 URs, 26 species (mean = 4.7 ± 1.1 URs per species); Hypertension: 116 URs, 26 species (mean = 4.5 ± 0.9 URs per species); Diarrhea: 68 URs, 42 species (mean = 1.6 ± 0.3 URs per species, highest species diversity); Flu: 162 URs, 35 species (mean = 4.6 ± 0.7 URs per species); UTI (Urinary Tract Infection): 63 URs, 17 species (mean = 3.7 ± 0.8 URs per species); Boils: 30 URs, 35 species (mean = 0.9 ± 0.2 URs per species); Bruises: 28 URs, 35 species (mean = 0.8 ± 0.2 URs per species). Error bars indicate standard error of mean URs per species for each condition. The high UR-to-species ratio for cough indicates cultural consensus on specific preferred species, while the high species-to-UR ratio for diarrhea suggests diverse treatment options with lower individual species preference.

In the study, wounds emerged as the most frequently cited condition, followed closely by fever, highlighting the community’s reliance on medicinal plants for managing both physical injuries and common ailments. These are followed by cough, diabetes, and hypertension, which indicate considerable concerns regarding respiratory, cardiovascular, and metabolic health. This is followed by diarrhea, indicating the prevalence of gastrointestinal conditions in the community. Next are flu and urinary tract infections (UTIs), which highlight the community’s management of seasonal illnesses through traditional remedies. While less frequently reported, conditions such as boils and bruises still reflect notable concerns over skin infections and minor injuries. These findings collectively suggest that the community frequently turns to medicinal plants to address a broad spectrum of infectious diseases, chronic health conditions, and injury-related concerns (Figure 6).

### Quantitative analyses

#### Use categories and use value (UV)

**FIGURE 7:**
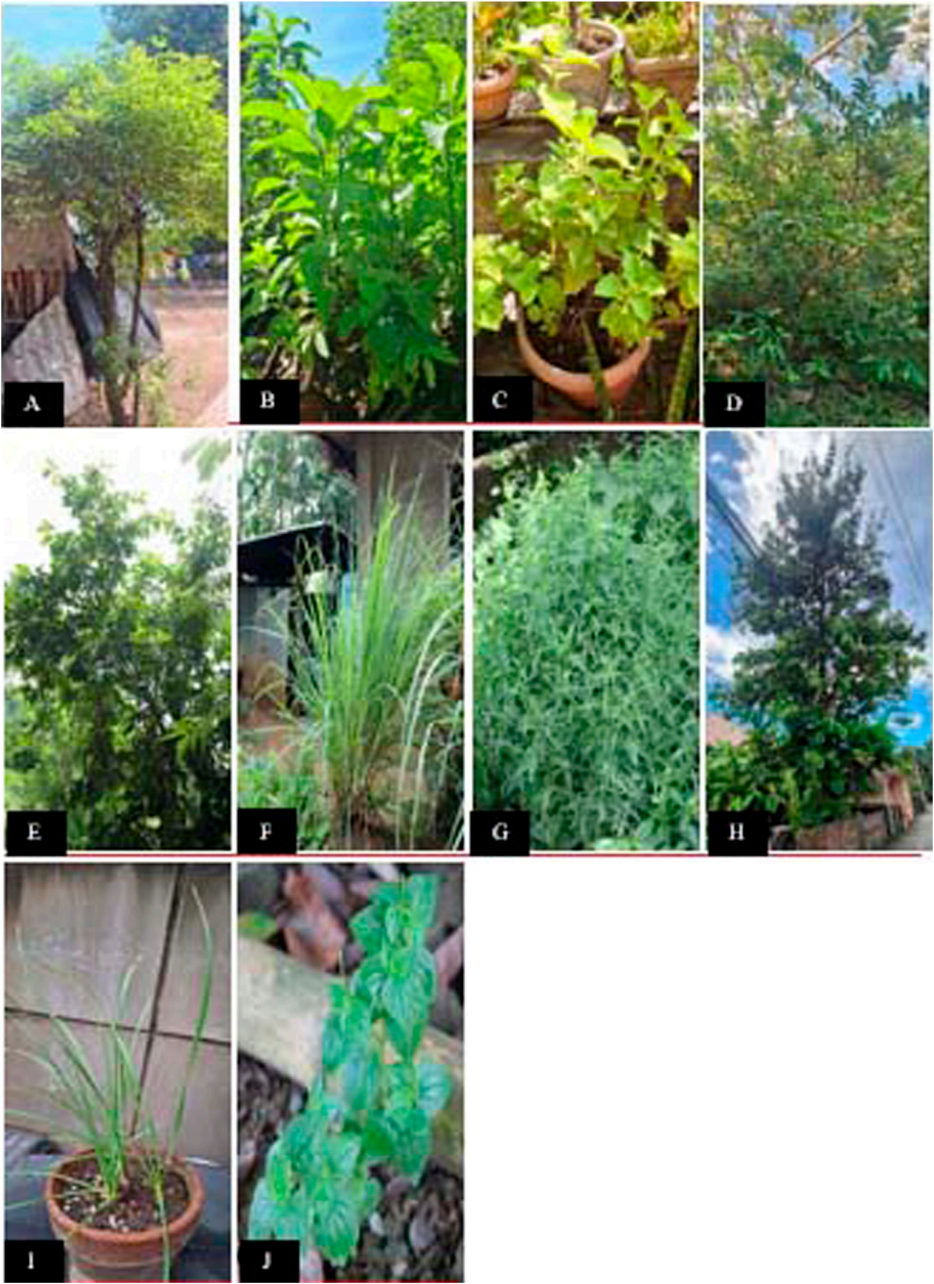
Photographs of the top 10 medicinal plant species ranked by Use Value (UV), showing growth habit and habitat (N = 252 informants). **(A)** *Vitex arvensis* Gentallan, Sengun and M.B. Bartolome (UV = 1.54, 178 informants, 387 URs, 14 conditions, 9 disease categories, RFC = 0.71, RI = 1.00) **(B)** *Blumea balsamifera* (L.); DC. (UV = 0.88, 66 informants, 223 URs, 12 conditions, 6 disease categories, RFC = 0.26); **(C)** *Coleus amboinicus* Lour. (UV = 0.66, 123 informants, 166 URs, 10 conditions, 5 disease categories, RFC = 0.49, RI = 0.62); **(D)** *Psidium guajava* L. (UV = 0.33, 45 informants, 83 URs, 12 conditions, 8 disease categories, RFC = 0.18); **(E)** *Annona muricata* L. (UV = 0.32, 48 informants, 81 URs, 14 conditions, 8 disease categories, RFC = 0.19, RI = 0.58); **(F)** *Cymbopogon citratus* (DC.) Stapf (UV = 0.31, 38 informants, 77 URs, 12 conditions, 7 disease categories, RFC = 0.15); **(G)** *Andrographis paniculata* (Burm.f.) Wall. ex Nees (UV = 0.30, 48 informants, 75 URs, 3 conditions, 3 disease categories, RFC = 0.19) **(H)** *Lagerstroemia speciosa* (L.); Pers. (UV = 0.20, 25 informants, 51 URs, 8 conditions, 7 disease categories, RFC = 0.10); **(I)** *Allium tuberosum* Rottler ex Spreng. (UV = 0.19, 29 informants, 47 URs, 6 conditions, 2 disease categories, RFC = 0.12) **(J)** *Peperomia pellucida* (L.); Kunth (UV = 0.14, 19 informants, 36 URs, 7 conditions, 4 disease categories, RFC = 0.08). UV calculated as total URs divided by total informants (N = 252). High UV indicates frequent and diverse use within the community. RFC (Relative Frequency of Citation) indicates proportion of informants citing each species. RI (Relative Importance) shown for top-ranked species.

Among the documented medicinal plants, *V. arvensis* Gentallan, Sengun and M.B. Bartolome emerged as the most versatile, being associated with nine disease categories and exhibiting the highest Use Value (UV) at 1.54, indicating its significant ethnobotanical relevance. *Annona muricata* L. and *P. guajava* L. followed closely, each being associated with eight disease categories, though their UVs were relatively low at 0.32 and 0.33, respectively. *Blumea balsamifera* (L.) DC (UV = 0.88) and *C. amboinicus* Lour. (UV = 0.66) had relatively high UVs despite being linked to fewer disease categories. Pictures of the top 10 medicinal plants with the highest UV are shown in Figure 7. The UV values, disease categories, and specific diseases associated with all the medicinal plant species in the study are shown in Supplementary Material 1.

#### Informant consensus factor (ICF)

The ICF measures agreement among informants on plant usage for specific disease categories. The highest ICF (1.00) was found in DC19 (Pregnancy, Childbirth, and the Puerperium), attributed solely to *S. macrophylla* King as an abortifacient (Use Report = 2). Despite high agreement, the low number of reports indicates niche or taboo usage. The second-highest ICF was in DC12 (0.95), where *V. arvensis* was the most cited for cough (Use Report = 157). This was followed by DC16 (0.91), where *B. balsamifera* was predominant for treating kidney stones (Use Report = 28). The lowest ICF (0.5) was observed in DC9, with *Calophyllum inophyllum* L. being cited for eye infections. This low score likely reflects fewer informants and limited plant consensus.

Comprehensive species information, including their scientific names, local uses, growth forms, conservation status, and results of other quantitative indices, is presented in Table 2, Supplementary Materials 3, 4.

**TABLE 2:** Medicinal plants in San Fernando, La Union with the highest number of disease categories, UV, RFC, and RI.

Rank	Scientific name	No. of DC’s	Scientific name	UV	Scientific name	RFC	Scientific name	RI
1	*Vitex arvensis* Gentallan, Sengun and M.B. Bartolome	9	*Vitex arvensis* Gentallan, Sengun and M.B. Bartolome	1.54	*Vitex arvensis* Gentallan, Sengun and M.B. Bartolome	0.71	*Vitex arvensis G*entallan, Sengun and M.B. Bartolome	1.00
2	*Annona muricata* L.	8	*Blumea balsamifera* (L.) DC.	0.88	*Coleus amboinicus* Lour.	0.49	*Coleus amboinicus* Lour.	0.62
3	*Cymbopogon citratus* (DC.) Stapf	8	*Coleus amboinicus* Lour.	0.66	*Blumea balsamifera* (L.) DC.	0.26	*Annona muricata* L.	0.58
4	*Psidium guajava* L.	8	*Psidium guajava* L.	0.33	*Annona muricata* L.	0.19	*Psidium guajava* L.	0.57
5	*Annona squamosa* L.	6	*Annona muricata* L.	0.32	*Andrographis paniculata* (Burm.f.) Wall. ex Nees	0.19	*Cymbopogon citratus* (DC.) Stapf	0.55
6	*Blumea balsamifera* (L.) DC.	6	*Cymbopogon citratus* (DC.) Stapf	0.31	*Psidium guajava* L.	0.18	*Blumea balsamifera* (L.) DC.	0.52
7	*Coleus amboinicus* Lour.	6	*Andrographis paniculata* (Burm.f.) Wall. ex Nees	0.30	*Cymbopogon citratus* (DC.) Stapf	0.15	*Moringa oleifera* Lam.	0.37
8	*Curcuma longa* L.	6	*Lagerstroemia speciosa* (L.) Pers.	0.20	*Allium tuberosum* Rottler ex Spreng.	0.12	*Annona squamosa* L.	0.36
9	*Moringa oleifera* Lam.	6	*Allium tuberosum* Rottler ex Spreng.	0.19	*Euphorbia hirta* L.	0.10	*Lagerstroemia speciosa* (L.) Pers.	0.35
10	*Ocimum tenuiflorum* L.	6	*Peperomia pellucida* (L.) Kunth	0.14	*Lagerstroemia speciosa* (L.) Pers.	0.10	*Ocimum tenuiflorum* L.	0.35

## Discussion

La Union remains poorly studied ethnobotanically. Only one previous study exists, conducted in Santol municipality (Ducusin, 2017), leaving most of the province undocumented despite its ecological diversity. In Ducusin’s study, 109 species from 20 genera and 15 families were documented for treating ailments under 13 disease categories, with respiratory and digestive disorders being the most common. In contrast, the present study identified 93 species from 86 genera and 45 families across 17 categories, with wounds as the most frequently cited ailment. The difference in the number of recorded species may be influenced by variations in sampling scope, ecological diversity, and the extent of ethnomedicinal knowledge shared by informants. Ducusin’s study involved only 40 informants, whereas the present study included 252, suggesting that a broader respondent base does not necessarily yield a higher number of species but may instead capture a more focused and widely shared body of medicinal knowledge. Environmental changes, land use, and reduced reliance on traditional healers may have also affected plant availability and recognition.

The most valued species in Ducusin’s study (UV = 1.00) included *V. arvensis*, *M. pudica*, and *P. guajava*, each widely used across different ailment categories. Similarly, *V. arvensis* also had the highest UV in the present study (UV = 1.54), reaffirming its consistent ethnomedicinal significance across La Union. In contrast, *P. guajava* (4^th^) and *M. pudica* (22^nd^) ranked lower, with UV values of 0.33 and 0.06, respectively. These differences may reflect variations in local health priorities, plant availability, and cultural perceptions of efficacy. Nevertheless, their continued documentation in both studies underscores their enduring cultural relevance and recognized therapeutic value in traditional healing practices.

Ducusin reported the highest informant consensus factor (ICF = 0.85) for genitourinary diseases, while the present study ranked this category (DC16) third (ICF = 0.91), after DC19 (Pregnancy, Childbirth, and the Puerperium; ICF = 1.00) and DC12 (Diseases of the Respiratory System; ICF = 0.95). The difference may be linked to variations in local health concerns, disease prevalence, and access to healthcare services. Both studies, however, shared a preference for leaves as the main plant part used, commonly prepared as decoctions due to their ease of preparation and high medicinal potency.

Medicinal plants in the community are mostly collected by locals within their surroundings or cultivated in home gardens. Many are grown for medicinal, culinary, or ornamental purposes. The cultivation of medicinal plants in backyard gardens holds significant ecological and conservation implications. This may help reduce harvesting pressure on wild populations and potentially support the sustainable use of medicinal plant resources. However, it may also lead to unintended consequences, such as decreased genetic diversity, habitat alteration, and competition with native flora (Schippmann et al., 2002; Mir et al., 2021; Mykhailenko et al., 2025). Knowledge transmission is deeply influenced by tradition and family. About 87.30% of informants learned from relatives and 31.35% from parents, emphasizing the strong role of oral tradition and hands-on experiences within families. Similar family-centered transmission patterns have been noted in other Philippine studies (Balangcod and Balangcod, 2011; Belgica et al., 2021). Community-based sharing also plays a role, with 77.3% of respondents acquiring plant knowledge through neighbors and informal exchanges, while modern channels, such as social media (7.94%) and friends (0.40%), play a minor role in knowledge transmission.

Healthcare patterns reveal the coexistence of multiple healthcare systems within the communities. In the study, 37% rely solely on traditional remedies, while 62% combine them with formal medical consultation. Respondents frequently turn to medicinal plants first, consulting physicians only if symptoms persist. This is primarily due to economic constraints and limited access to healthcare. This highlights the continued practical and cultural relevance of medicinal plants in the face of modernization. Only 0.4% of the participants stated that they sought the help of both a mystic healer or *albularyo* and a medical doctor, suggesting that reliance on traditional healers is relatively rare in the community.

Most medicinal plants documented were not listed in the IUCN Red List of Threatened Species (IUCN, 2024), consistent with other findings from other ethnobotanical studies in the Philippines (Dapar et al., 2020; Cordero et al., 2023; 2025) and abroad (Dessie and Amsalu, 2024; Ghafouri et al., 2025). The only endangered species identified was *S. macrophylla* King, a high-value hardwood regulated under CITES Appendix II due to overexploitation (Barstow and Negrão, 2023; CITES, 2024). Ironically, it is also among the most invasive trees introduced for reforestation in the Philippines (Baguinon et al., 2003; Coracero, 2023). The prevalence of naturalized species reflects trends reported in other ethnobotanical studies (Dapar and Amoroso, 2022; Cordero et al., 2023) and indicates a strategic adaptation by local communities that prioritize accessible, resilient, and easily cultivated plants for traditional healing practices.

One endemic species (*V. arvensis* Gentallan, Sengun and M.B. Bartolome) was identified among the 26 native species documented. *V. arvensis* is one of the most well-known herbal medicines in the Philippines and is listed among the scientifically validated medicinal plants by the Department of Health (PITAHC, 2025). It typically grows along roadsides and streambanks (Boy et al., 2018) and is commonly found in backyard gardens in the community. Most of the documented medicinal plants are naturalized species. This observation is consistent with findings from other ethnobotanical studies in the Philippines (Dapar and Amoroso, 2022; Cordero et al., 2023) and globally (Cadena-González et al., 2013; González-Ball et al., 2022). The dominance of naturalized species over native ones may be attributed to their availability and adaptability. Naturalized species often thrive in a wide range of environmental conditions, making them more accessible for medicinal use. Their ability to spread and establish in diverse ecosystems increases their likelihood of incorporation into traditional healing practices (Hart et al., 2017). Moreover, they are often easy to propagate and suitable for cultivation in home gardens and urban areas (González-Ball et al., 2022).

Among the 45 plant families recorded, the Fabaceae family had the highest representation, consistent with previous findings from other ethnobotanical studies in the Philippines (Omac et al., 2021; Cabugatan et al., 2022; Cordero et al., 2025) and abroad (Nyirenda and Chipuwa, 2024; Anwar et al., 2024). Its species were primarily used to treat digestive disorders and are rich in bioactive compounds such as flavonoids, terpenoids, and alkaloids (Maroyi, 2023), which exhibit anticancer (Abdulqawi et al., 2021), antidiabetic (Qian et al., 2022), antimicrobial (Obakiro et al., 2021), and anti-inflammatory properties (Oladeji et al., 2021).

Leaves were the most frequently used plant part because they regenerate quickly and can be collected without harming the plant, making them a renewable and sustainable resource for medicinal use. Likewise, they are rich in bioactive secondary metabolites (alkaloids, flavonoids, tannins, and essential oils), which provide therapeutic properties such as anti-inflammatory, antimicrobial, and analgesic effects (Sharma et al., 2017; Amat-ur-Rasool et al., 2020; Kumar et al., 2021a; 2021b). The most common method of preparation was decoction, typically administered orally as herbal teas or tonics, similar to other studies in the Philippines (Bersamin et al., 2021; Cordero and Alejandro, 2021) and abroad (Ali et al., 2017; Lal et al., 2023). Other uses include topical application for washing or bathing, and in some cases, steam inhalation (locally referred to as *suob*).

*Vitex arvensis* Gentallan, Sengun and M.B. Bartolome emerged as the most culturally significant medicinal plant in this study, ranking highest across multiple ethnobotanical indices (UV = 1.54, RFC = 0.71, RI = 1.00, ICF = 0.95 for respiratory diseases). This is confirmed through quantitative methods, which have long been observed in practice across northern Luzon but have never been systematically measured. Its widespread use and high cultural consensus suggest strong community trust in its therapeutic efficacy, though ethnobotanical significance does not automatically equate to pharmacological efficacy without biochemical validation (Heinrich et al., 2009). Nevertheless, the documented uses of *V. arvensis* in this study, particularly for respiratory conditions (cough, asthma, flu), inflammatory conditions (rheumatism, fever), and gastrointestinal ailments (diarrhea), align with phytochemical evidence from related *Vitex* species. Studies have isolated bioactive compounds, including flavonoids (casticin, vitexicarpin), iridoid glycosides (agnuside, aucubin), terpenoids, and phenolic acids from various *Vitex* species, demonstrating anti-inflammatory, analgesic, antimicrobial, antioxidant, and bronchodilatory properties *in vitro* and *in vivo* (Khan et al., 2017; Kamal et al., 2021; Garg et al., 2024). In the Philippines, *V. arvensis* has been scientifically validated and listed by the Department of Health among ten priority herbal medicines (PITAHC, 2025), with clinical evidence supporting its use for cough and asthma (Khan et al., 2015). However, further pharmacognostic research specific to La Union populations is warranted to confirm the biochemical basis of locally reported uses and to assess potential chemotype variation across geographical populations.

The high ethnobotanical indices for *V. arvensis* may also reflect accessibility factors, as the species grows readily in backyard gardens and along roadsides (Boy et al., 2018), thereby reducing harvesting effort compared to forest species. This availability likely reinforces its cultural prominence through regular use and intergenerational transmission. The combination of pharmacological potential, cultural familiarity, and ecological accessibility positions *V. arvensis* as a priority candidate for conservation, sustainable cultivation, and further ethnopharmacological investigation in the region.

*Coleus amboinicus* Lour. ranked third in UV, second in RFC, and second in RI, emphasizing its significant role in traditional knowledge and daily use. Although it did not rank in the top three for the number of disease categories treated, it remains highly valued due to its accessibility and multiple medicinal applications. Informants noted that *C. amboinicus* is primarily used for the treatment of respiratory diseases such as asthma, bronchitis, cough, and sore throat. Typically, 1–2 tbsp of extracted juice from crushed leaves are taken orally 2–3 times a day. In some cases, locals boil the leaves in water, and 1 cup of the decoction is taken orally. Consistent with other ethnobotanical studies in the Philippines, the use of *C. amboinicus* for respiratory diseases has also been documented in Camarines Sur (Cerio, 2024), Iligan City (Olowa and Demayo, 2015), and Iloilo City (Cordero et al., 2023). Similarly, findings have been reported internationally, including in Brazil (Cerqueira et al., 2020), Taiwan (Huang et al., 2022), India (Ramar et al., 2023), and Indonesia (Bria et al., 2022), reflecting the widespread recognition of *C. amboinicus* as a traditional remedy for respiratory conditions. Furthermore, *C. amboinicus* is known to contain two major bioactive compounds, carvacrol and thymol (Arumugam et al., 2016; Karadayı et al., 2020), which makes it an excellent expectorant commonly used to treat respiratory disorders. These compounds exhibit bronchodilatory properties that help relax airway smooth muscles and inhibit microbial growth in the respiratory tract (Jaramillo-Colorado et al., 2022; Piasecki et al., 2023; Phattayanon et al., 2024; Can Baser et al., 2025). In addition, other compounds such as linalool, terpinene, geraniol, and eugenol contribute antioxidant and anti-inflammatory activities that further alleviate airway irritation and promote mucosal recovery (Phattayanon et al., 2024).

*Blumea balsamifera* (L.) DC. ranked second in UV and third in RFC but was sixth in both RI and the number of disease categories. Its absence from the top three RI rankings implies that while it has multiple uses, its overall ethnomedicinal impact may be lower than that of plants with both a high frequency of use and broader applications. Compared to *C. amboinicus*, which is more frequently cited, *B. balsamifera* appears to be more diverse in its applications but is less commonly used. *B. balsamifera* is commonly used by locals to treat genitourinary diseases like kidney stones, urinary tract infections, dysuria, and dysmenorrhea. The leaves are usually boiled, and 1–2 cups of the decoction are taken orally 2–3 times a day. It is also known to treat other diseases such as cough, colds, fever, flu, and *pasma*, where the leaves are boiled and the warm decoction is used for a steam bath. The decoction is sometimes made together with other medicinal plants such as *V. arvensis*, *P. guajava,* and *C. citratus*. In addition to these ailments, *B. balsamifera* has been documented in several ethnobotanical studies across the Philippines for a range of therapeutic purposes. It has been cited for the treatment of hypertension in Camarines Sur (Cerio, 2024), post-partum care in Zamboanga (Madjos and Ramos, 2021a), and arthritis in Bukidnon (Naive et al., 2021). Comparable uses have also been reported in other parts of Asia, where the species is employed for hypertension management in Thailand (Sumridpiem et al., 2025), post-partum recovery in Central Laos (Phengmala et al., 2024), and arthritis treatment in China (Zheng and Xing, 2009). These consistent reports across different cultural and geographic contexts highlight the widespread recognition of *B. balsamifera* as an important medicinal resource in traditional healthcare systems throughout Asia. The use of *B. balsamifera* is supported by the presence of bioactive compounds such as flavonoids which contributes to genitourinary health and wound healing; chalcones which may aid in hypertension and other cardiovascular conditions; and aldehydes, which exhibits anti-inflammatory activity (Widhiantara and Jawi, 2021).

*Annona muricata* L. ranked fifth in UV, second in the number of disease categories it treats, fourth in RFC, and third in RI. This suggests that it holds significant therapeutic value for specific applications but is less frequently cited than leading species like *V. arvensis* and *C. amboinicus. A. muricata* has also been used by the locals to treat a wide range of diseases like dysentery, flu, diabetes, hypertension, cough, urinary tract infection, and cancer (Madjos and Ramos, 2021b; Montero and Geducos, 2021; Nuneza et al., 2021). The leaves are often boiled, and the resulting decoction is taken orally, while the fruit is eaten raw. A. muricata contains bioactive compounds, including acetogenins, phenolics, alkaloids, flavonoids, carbohydrates, saponins, and tannins. These compounds are responsible for the antioxidant, anticancer, antibacterial, antiviral, antiulcer, and antidiarrheal properties of *A. muricata* (Mesquita et al., 2021; Mutakin et al., 2022; Ruiz-López et al., 2025; Igiehon et al., 2025).

*Psidium guajava* L. ranked third in the number of disease categories, fourth in UV, and fourth in RI. Although it was sixth in RFC, suggesting it is less frequently mentioned by respondents, it remains a key ethnobotanical species. It was cited by locals to be a good remedy for boils, bruises, and wounds (Madjos and Ramos, 2021b; Mangali et al., 2021). Leaves are sometimes boiled, and the decoction is used to wash affected body parts. The leaves can also be pounded into a paste and applied as a poultice. *P. guajava* is also used to treat other ailments such as diabetes, hypertension, colds, cough, and even diarrhea (Antonio and Tuason, 2022; Cordero et al., 2023; Saro et al., 2022). The locals prepare the treatment by boiling the leaves and drinking the decoction. *P. guajava* is also a good source of bioactive compounds like catechin, polyphenols, gallic acid, flavonoids, kaempferol, and guaijaverin, among others. This gives *P. guajava* several medicinal properties, such as anticancer, antidiabetic, antimicrobial, antioxidant, anti-inflammatory, and antidiarrheal properties (Jiang et al., 2020; Kumar et al., 2021b; Wani and Uppaluri, 2022; Lee et al., 2024; Huynh et al., 2025).

Location was the only factor that significantly affected the number of medicinal plants people knew. The Kruskal-Wallis test showed clear differences among barangays (p < 0.001). People in Bacsil knew an average of 8.1 species, while those in Abut knew 8.4 species, and residents of Saoay knew only 5.2 species on average. Gender, marital status, occupation, education level, and age did not make a difference. Men and women knew essentially the same number of species (7.2 vs. 7.3). Older people knew no more than younger ones. Education level had no effect. This suggests that medicinal plant knowledge is shared widely across the community, rather than being concentrated in certain groups.

The geographic pattern makes sense when viewed in the context of the physical landscape. Bacsil and Abut sit at higher elevations with more vegetation and fewer people, allowing for a greater variety of plant species to thrive in the area. These barangays are also farther from hospitals (15.8 km and 11.1 km) with steep, rough roads that make travel difficult. Saoay is lower, more developed, and closer to town (8.3 km), so people have easier access to doctors and pharmacies. Similar geographic patterns have been observed in other Philippine studies (Balangcod and Balangcod, 2011; Cordero et al., 2023). However, some international studies have reported age or gender effects, which may be attributed to variations in cultural practices related to knowledge transmission.

These findings have practical implications for conservation efforts. The successful knowledge transmission across age groups is encouraging, suggesting that traditional practices remain culturally valued. However, the geographic concentration of knowledge in higher-elevation barangays creates vulnerability, as localized environmental or social changes could result in significant knowledge loss. Conservation strategies should therefore prioritize both maintaining ecological conditions that support plant diversity and creating knowledge-sharing networks between communities at different elevations. The equal distribution across genders indicates that both men and women can effectively serve as key informants and educators in preservation programs.

### Comparative regional perspectives and ecological implications

The documented species richness (93 species) in this study falls within the mid-range of Philippine ethnobotanical studies, suggestinOngg moderate knowledge retention in semi-urban agricultural contexts. Compared to indigenous communities like Ati groups (106–142 species; Cordero et al., 2020; Cordero and Alejandro, 2021), Panay Bukidnon (127 species; Cordero et al., 2022a), Kalanguya (125 species; Balangcod and Balangcod, 2011), landlocked agricultural communities in San Fernando demonstrate considerable ethnobotanical knowledge despite greater modernization pressures. This aligns with findings from other Southeast Asian transitional communities, where medicinal plant knowledge persists even under healthcare modernization, provided that: 1. geographic isolation maintains dependence on local resources, 2. agricultural livelihoods preserve plant cultivation traditions, and 3. intergenerational knowledge transmission remains intact (Phumthum et al., 2020; Widodo et al., 2023).

The dominance of naturalized species (56%, n = 43) over native species (34%, n = 26) in our study reflects a global pattern documented across Southeast Asia (Cadena-González et al., 2013; González-Ball et al., 2022), Nepal (Paudel et al., 2021), and Africa (Maroyi, 2023). This “introduced species bias” may result from: 1. ecological adaptability where naturalized species tolerate disturbed habitats and are easily cultivated in home gardens, 2. availability bias where readily accessible species become preferentially incorporated into medicinal systems, and 3. knowledge exchange where globalization facilitates sharing of naturalized medicinal plants across cultural boundaries (Hart et al., 2017; González-Ball et al., 2022). However, the continued use of native species (particularly *V. arvensis*, *A. catechu*) demonstrates that cultural preference and perceived efficacy can override availability factors for certain conditions.

From a conservation perspective, several findings warrant attention. First, the presence of *S. macrophylla* (IUCN Endangered) in local medicine highlights potential conflicts between conservation policy and traditional use. Although *S. macrophylla* is widely cultivated and even invasive in the Philippines (Coracero, 2023), international trade restrictions (CITES Appendix II) may inadvertently affect local access. Second, the heavy reliance on cultivated and naturalized species (76% of documented flora) suggests reduced harvesting pressure on wild native populations, which is a positive conservation outcome. However, this also indicates a potential erosion of knowledge regarding wild, native medicinal plants, as younger generations increasingly rely on garden-cultivated, introduced species. Third, the concentration of knowledge in higher-elevation barangays (Bacsil, Abut) suggests that these communities serve as repositories of traditional ecological knowledge and may require targeted conservation interventions. If development pressures continue, knowledge loss in these communities could be irreversible.

The equal distribution of knowledge across age groups, unlike patterns documented in rapidly urbanizing areas where younger generations show reduced ethnobotanical knowledge (Reyes-García et al., 2013; Srithi et al., 2009), indicates active intergenerational transmission in these communities. This resilience may stem from the persistent relevance of medicinal plants in daily healthcare, as evidenced by 37% of informants relying exclusively on herbal remedies and 62% combining traditional and biomedical care. However, the lower knowledge in Saoay (closer to urban centers, better healthcare access) foreshadows potential erosion patterns if socioeconomic development continues without deliberate cultural preservation efforts.

## Conclusion

This study provides a comprehensive ethnobotanical documentation of 93 medicinal plant species used by landlocked agricultural communities in San Fernando, La Union, filling a critical knowledge gap in an understudied Philippine province. Quantitative ethnobotanical analysis identified *V. arvensis*, *C. amboinicus*, *B. balsamifera*, *A. muricata*, and *P. guajava* as culturally significant species warranting further investigation. Significant geographic variation in medicinal plant knowledge (p < 0.001) underscores the influence of elevation, accessibility, and ecological context on traditional knowledge systems, while the absence of variation across gender, age, education, and occupation suggests broad community-level knowledge distribution.

### Distinction between ethnobotanical documentation and pharmacological validation

It is critical to emphasize that this study documents ethnobotanical knowledge*,* including the cultural uses, preparation methods, and perceived therapeutic value of medicinal plants within a specific community. While we contextualize findings with existing phytochemical and pharmacological literature where available, ethnobotanical documentation does not constitute pharmacological validation. The reported medicinal uses reflect traditional knowledge systems and cultural consensus, but biochemical efficacy, safety profiles, optimal dosages, and mechanisms of action require rigorous pharmacognostic, phytochemical, toxicological, and clinical research. Our study provides a foundational dataset to prioritize species for such investigations, but claims of therapeutic efficacy must be substantiated through controlled laboratory and clinical studies before any biomedical applications can be recommended.

### Study limitations

Several limitations should be acknowledged. First, the reliance on retrospective recall introduces potential memory bias, particularly for infrequently used species. Second, while data saturation was achieved for species richness, saturation of preparation methods and dosage information may be incomplete. Third, the absence of phytochemical analysis in this study limits our ability to assess intraspecific chemical variation or verify the presence of active compounds in locally harvested specimens. Fourth, although two taxonomic experts verified plant identifications, molecular barcoding would provide additional confirmation for morphologically cryptic species. Finally, our sampling design (three barangays, one municipality) limits generalizability across La Union Province or the broader Ilocos Region; however, it provides a replicable methodological framework for future comparative studies.

### Future research directions and conservation implications

Future research should: 1. conduct phytochemical screening and bioactivity assays on priority species identified in this study, particularly *V. arvensis*, while using *C. amboinicus*, and *B. balsamifera*, as reference taxa for comparative analyses; 2. expand ethnobotanical surveys to coastal and near-coastal barangays to assess ecological gradient effects on medicinal plant diversity; 3. investigate preparation methods and dosage regimens through participatory research with experienced practitioners to clarify how traditional techniques affect bioactive compounds efficacy and reflect cultural preferences; 4. document seasonal variation in plant use and availability; and 5. assess cultivation potential and sustainable harvesting practices for priority species.

Future studies should examine preparation methods and dosage regimens with experienced practitioners to clarify how traditional techniques affect bioactive compound efficacy and reflect cultural preferences.

From a conservation perspective, the integration of traditional knowledge with scientific validation can strengthen both cultural preservation and biodiversity conservation goals. Community-based conservation programs should prioritize: 1. establishment of medicinal plant gardens in schools and health centers to maintain living repositories, 2. documentation of preparation methods through multimedia platforms accessible to younger generations, 3. sustainable cultivation training for frequently used species to reduce wild harvesting pressure, and 4. integration of traditional knowledge into local health systems through collaborative partnerships between traditional practitioners and healthcare providers.

In conclusion, this study demonstrates that landlocked agricultural communities in San Fernando, La Union, maintain substantial ethnobotanical knowledge despite socioeconomic modernization. Geographic isolation, agricultural livelihoods, and active intergenerational transmission sustain this knowledge, but urbanization and healthcare modernization pose ongoing threats. Systematic documentation, scientific validation, and community-based conservation are essential to preserve this biocultural heritage for future generations while ensuring its safe and effective integration into contemporary healthcare systems.

## Data Availability

The original contributions presented in the study are included in the article/Supplementary Material, further inquiries can be directed to the corresponding authors.
