# Supplementary material for: Ethnobotanical survey and quantitative assessment of medicinal plants in landlocked communities of San Fernando, La Union, Philippines
Source: Front Pharmacol. 2025 Nov 13;16:1670496. doi: 10.3389/fphar.2025.1670496 (PMC12657414; doi:10.3389/fphar.2025.1670496)
Supplement: Supplementary file 1 [file Supplementaryfile1.docx]

**References (Supplementary Materials):**

Albuquerque, U.P., Lucena, R.F.P., Monteiro, J.M., Florentino, A.T.N., and Almeida, C. de F.C.B.R. (2006). Evaluating Two Quantitative Ethnobotanical Techniques. *Ethnobotany Research and Applications*, *4*, 051–060.

Albuquerque, U.P., Lucena, R.F.P., Cunha, L.V.F.C. (2014). Methods and Techniques in Ethnobiology and Ethnoecology. Humana Press, New York.

Bernard, H.R. (2017). Research Methods in Anthropology: Qualitative and Quantitative Approaches, 6th ed. Rowman & Littlefield, Lanham, MD.

Cordero, C.S., Meve, U., and Alejandro, G.J.D. (2022b). Quantitative ethnobotanical documentation of medicinal plants used by the indigenous Ati tribes in Panay Island, Philippines. *Malaysian Journal of Sustainable Environment (MySE)*, *9*(2), 143-170.

Daniel, W.W. (1999). Biostatistics: A Foundation for Analysis in the Health Sciences, 7th ed. John Wiley & Sons, New York.

Dapar, M.L.G., Alejandro, G.J.D., Meve, U., and Liede-Schumann, S. (2020b). Quantitative ethnopharmacological documentation and molecular confirmation of medicinal plants used by the Manobo tribe of Agusan del Sur, Philippines. *J. Ethnobiol. Ethnomed.*, 16, 1-60.

Friedman, J., Yaniv, Z., Dafni, A., and Palewitch, D. (1986). A preliminary classification of the healing potential of medicinal plants, based on a rational analysis of an ethnopharmacological field survey among Bedouins in the Negev Desert, Israel. *Journal of ethnopharmacology*, *16*(2-3), 275-287. <https://doi.org/10.1016/0378-8741(86)90094-2>

Heinrich, M., Ankli, A., Frei, B., Weimann, C., Sticher, O. (1998). Medicinal plants in Mexico: Healers' consensus and cultural importance. Soc. Sci. Med. 47, 1859-1871.

Leonti, M., Casu, L. (2013). Traditional medicines and globalization: current and future perspectives in ethnopharmacology. Front. Pharmacol. 4, 92.

Martin, G.J. (2004). Ethnobotany: A Methods Manual. Earthscan, London.

Ong, H.G., and Kim, Y.D. (2014). Quantitative ethnobotanical study of the medicinal plants used by the Ati Negrito indigenous group in Guimaras island, Philippines. *J. Ethnopharmacol.*, 157, 228-242.

Phillips, O., Gentry, A.H. The useful plants of Tambopata, Peru: I. Statistical hypotheses tests with a new quantitative technique. *Econ Bot* **47**, 15–32 (1993). <https://doi.org/10.1007/BF02862203>

Phillips, O., Gentry, A.H. The useful plants of Tambopata, Peru: II. Additional hypothesis testing in quantitative ethnobotany. *Econ Bot* **47**, 33–43 (1993). <https://doi.org/10.1007/BF02862204>

Soberón, J., Llorente, J. (1993). The use of species accumulation functions for the prediction of species richness. Conserv. Biol. 7, 480-488.

Tardío, J., and Pardo-de-Santayana, M. (2008). Cultural importance indices: A comparative analysis based on the useful wild plants of Southern Cantabria (Northern Spain). *Econ. Bot.*, 62(1), 24–39.

Trotter, R.T. and Logan, M.H. (1986). Informant Consensus: A new approach for identifying potentially effective medicinal plants. In N.L. Etkin (Ed.), Plants in indigenous medicine and diet: Biobehavioral approach (pp. 91-112). Redgrave Publishing Company, Bedford Hills. New York.

WHO. (2024). International classification of diseases (ICD). ICD-11 for mortality and morbidity statistics (version: 01/2025). World Health Organization. https://icd.who.int/browse/2025-01/mms/en (Accessed 20/02/2025).
