# Supplementary material for: Ethnobotanical survey and quantitative assessment of medicinal plants in landlocked communities of San Fernando, La Union, Philippines
Source: Front Pharmacol. 2025 Nov 13;16:1670496. doi: 10.3389/fphar.2025.1670496 (PMC12657414; doi:10.3389/fphar.2025.1670496)
Supplement: Supplementary file 2 [file Table1.docx]

**Supplementary Materials:**

**Supplementary Material 1.**

***Sample Size Determination and Justification***

The target sample size was calculated using the formula for finite population proportion (Daniel, 1999): n = (Z²pq)/(d²), where Z = 1.96 (95% confidence level), p = 0.5 (maximum variability), q = 0.5, and d = 0.05 (margin of error). For the combined population of 2,386 residents across the three barangays, the minimum required sample was calculated as:

n = (1.96² × 0.5 × 0.5) / (0.05²) = 384

Applying the finite population correction: n_adj = n / (1 + (n-1)/N) = 384 / (1 + 383/2,386) = 331

Our achieved sample of 252 informants represents 10.6% of the total population and 76.1% of the statistically required minimum. Given the clustered nature of ethnobotanical knowledge (Bernard, 2017) and the achievement of data saturation (detailed below), this sample size provides adequate statistical power for ethnobotanical analysis.

The 10% sampling fraction was deliberately exceeded in smaller barangays to ensure proportional representation: Barangay Abut (67/642 = 10.4%), Barangay Bacsil (79/722 = 10.9%), and Barangay Saoay (106/1,022 = 10.4%). This approach balances statistical requirements with practical constraints of house-to-house surveys in geographically dispersed communities (Martin, 2004).

***Assessment of Data Saturation and Informant Reliability***

Data saturation was assessed using the species accumulation curve approach (Soberón and Llorente, 1993), where cumulative species richness was plotted against the number of informants interviewed. Saturation was considered achieved when: (1) no new species were reported in three consecutive sets of 10 informants, and (2) the species accumulation curve reached an asymptote with <1% increase in species richness over the final 20% of interviews. In this study, saturation was observed at interview 215, after which 37 additional interviews yielded no new species (Figure 2).

Informant reliability was assessed through multiple validation mechanisms:

1. **Internal consistency checks:** For species cited by multiple informants (n = 64 species), use reports were cross-validated for consistency in plant part, preparation method, and medicinal indication. Concordance rates exceeded 85% for the 10 most frequently cited species.
2. **Medical diagnosis verification:** For informants reporting chronic conditions (diabetes, hypertension, asthma, arthritis, kidney disease; n = 147, 58.3%), medical diagnosis was confirmed through documentary evidence (prescription records, health center records) or direct consultation verification. This approach ensured reported ailments were based on clinical diagnosis rather than self-perception, reducing self-diagnosis bias (Leonti and Casu, 2013).
3. **Taxonomic verification:** All plant species mentioned by informants were collected, photographed, and herbarium-verified to ensure accurate species identification, eliminating potential misidentification errors common in vernacular name-based studies.

These validation procedures enhance data reliability and reduce common biases in ethnobotanical surveys (Tardío and Pardo-de-Santayana, 2008; Albuquerque et al., 2014).
