# Supplementary material for: Ethnobotanical survey and quantitative assessment of medicinal plants in landlocked communities of San Fernando, La Union, Philippines
Source: Front Pharmacol. 2025 Nov 13;16:1670496. doi: 10.3389/fphar.2025.1670496 (PMC12657414; doi:10.3389/fphar.2025.1670496)
Supplement: Supplementary file 3 [file Table2.docx]

**Supplementary Material 2:**

***Data validation and statistical assumptions***

Prior to calculating ethnobotanical indices, data distributions were assessed for normality using the Shapiro-Wilk test (p < .05 indicates non-normality). The number of species cited per informant showed significant deviation from normality (W = 0.917, p < .001), justifying the use of non-parametric tests (Mann-Whitney U, Kruskal-Wallis H) for group comparisons.

For Informant Consensus Factor (ICF) calculations, disease categories with fewer than 5 use reports were excluded from analysis to ensure statistical validity (Heinrich et al., 1998). Of the 18 disease categories identified, 17 met this threshold. Use Value (UV) and Relative Importance (RI) indices were calculated separately for each barangay before pooling to account for differing informant counts (Barangay Abut: n = 67; Bacsil: n = 79; Saoay: n = 106). Pooled indices were weighted by barangay sample size to prevent over-representation bias from larger communities. Sensitivity analyses confirmed that index rankings were robust across weighted and unweighted calculations (Spearman's ρ > 0.95 for the top 20 species).

To address potential pseudo-replication, each informant-species-ailment combination was treated as a single use report (UR), regardless of how many times an informant mentioned the same use. This conservative approach prevents inflated use values from verbose informants (Albuquerque et al., 2006; Phillips and Gentry, 1993a,b).

***Unit of Analysis and Data Structure***

All quantitative analyses employ use reports (URs) as the fundamental unit of analysis, defined as a single citation of a plant species for a specific ailment by an individual informant (Trotter and Logan, 1986). Multiple URs from the same informant for different ailments are counted independently, while repeated mentions of the same species-ailment combination by the same informant are counted only once to prevent pseudo-replication (Phillips and Gentry, 1993a,b). Ethnobotanical indices (UV, RFC, RI, FL, ICF) are calculated at the species level but derived from individual-level use reports, ensuring statistical independence of observations. Sociodemographic analyses use individual informants as the unit of analysis, with the number of species cited per informant as the dependent variable (mean ± SD reported throughout).

***Disease Categories and Computation of Other Indices:***

***Disease Categories (DCs and Use Categories (UCs)***

DCs are broad classifications based on the International Classification of Diseases (ICD), which organizes diseases by cause, symptoms, or the body system affected. This global system, maintained by the World Health Organization, is widely used for research, clinical practice, and policy-making purposes. UCs, on the other hand, were employed to classify medicinal plants based on the types of health conditions or diseases they are used to treat, in accordance with the ICD-11, which recognizes 26 major categories (WHO, 2024).

***Relative Frequency of Citation (RFC)***

The RFC was used to measure the frequency with which a species is mentioned by informants. It is calculated using the formula RFC = FC/N, where FC is the number of informants who mentioned the species, and N is the total number of informants participating in the study. Higher RFC values indicate species that are more commonly used or widely recognized within the community (Tardío and Pardo-de-Santayana, 2008).

***Relative Importance Index (RI)***

The RI was used to account for both the frequency of citation and the number of use categories for each species. It is calculated as RI = (RFC + RNU)/2; where: RFC = FC/FC and RNU = NU/NU

Here, FC is the number of informants citing the species, FC is the highest citation count recorded for any species, NU is the number of use categories the species falls under, and NU is the highest number of use categories among all species. RI values closer to 1 signify broad use and high consensus among informants (Tardío and Pardo-De-Santayana, 2008; Cordero et al., 2022b).

***Fidelity Level (FL)***

The FL was used to assess the cultural importance of a plant based on how consistently it is used for specific ailments. It is calculated using FL (%) = (I / I) × 100, where I is the number of informants who cited the plant species for a specific ailment and I is the number of informants who mentioned the species for any use (Friedman et al., 1986; Ong and Kim, 2014; Dapar et al., 2020b). A high FL indicates that the plant is widely recognized for a specific treatment.
