# Supplementary material for: Ethnobotanical survey and quantitative assessment of medicinal plants in landlocked communities of San Fernando, La Union, Philippines
Source: Front Pharmacol. 2025 Nov 13;16:1670496. doi: 10.3389/fphar.2025.1670496 (PMC12657414; doi:10.3389/fphar.2025.1670496)
Supplement: Supplementary file 4 [file Table3.docx]

Supplementary Material 3. Medicinal plants used by the locals of the City of San Fernando, La Union

| Family | Scientific Name | Common Names | †Origin | ⸸IUCN | ‡Growth Forms | ⁑Plant Parts | Disease/Purpose | *DC | Preparation and administration | UR | UV | RFC | RI |
| --- | --- | --- | --- | --- | --- | --- | --- | --- | --- | --- | --- | --- | --- |
| Acanthaceae | *Andrographis paniculata*(Burm.f.) Wall. ex Nees | King of Bitters (Eng.), Sinta (Tag.), Serpentina (Ilk.) | Nu | NL | H | L | Diabetes | 5 | Boil or infuse leaves in hot water and drink decoction | 75 | 0.30 | 0.19 | 0.30 |
|  |  |  |  |  |  | L | Hypertension | 11 | Boil or infuse leaves in hot water and drink decoction |  |  |  |  |
|  |  |  |  |  |  | L | Cough | 12 | Boil or infuse leaves in hot water and drink decoction |  |  |  |  |
| Amaryllidaceae | *Allium sativum* L. | Garlic (Eng.), Bawang (Tag., Ilk.) | CNu | NL | H | Bu | Hypertension | 11 | Eat raw cloves | 12 | 0.05 | 0.04 | 0.19 |
|  |  |  |  |  |  | Bu | Headache | 21 | Pound cloves into a paste and massage onto the temple or forehead; Boil crushed cloves and drink decoction |  |  |  |  |
|  |  |  |  |  |  | Bu | Bruises | 22 | Pound cloves into a paste and apply as poultice |  |  |  |  |
|  |  |  |  |  |  | Bu | Sprain | 22 | Pound cloves into a paste and apply as poultice |  |  |  |  |
| Amaryllidaceae | *Allium tuberosum* Rottler ex Spreng. | Chinese chives (Eng.), Kuchay (Tag.), Kutsay (Ilk.) | CNu | NL | H | L | Boils | 1 | Pound leaves into a paste and apply as poultice | 47 | 0.19 | 0.12 | 0.20 |
|  |  |  |  |  |  | L | Bruises | 22 | Pound leaves into a paste and apply as poultice |  |  |  |  |
|  |  |  |  |  |  | L | Bumps | 22 | Pound leaves into a paste and apply as poultice |  |  |  |  |
|  |  |  |  |  |  | L | Burns | 22 | Pound leaves into a paste and apply as poultice |  |  |  |  |
|  |  |  |  |  |  | L | Sprain | 22 | Pound leaves into a paste and apply as poultice |  |  |  |  |
|  |  |  |  |  |  | L | Wounds | 22 | Pound leaves into a paste and apply as poultice |  |  |  |  |
| Anacardiaceae | *Anacardium occidentale* L. | Cashew (Eng.), Kasuy (Tag., Ilk.) | Nu | LC | T | Ba | Diabetes | 5 | Boil bark and drink decoction | 6 | 0.02 | 0.02 | 0.18 |
|  |  |  |  |  |  | Fr | Cough | 12 | Eat raw fruit |  |  |  |  |
|  |  |  |  |  |  | L | Toothache | 13 | Boil leaves and drink decoction; Pound leaves into a paste and apply as poultice |  |  |  |  |
| Anacardiaceae | *Mangifera indica* L. | Mango (Eng.), Manga (Tag., Ilk.) | Nu | DD | T | L, Ba | Hypertension | 11 | Boil leaves or bark and drink decoction | 10 | 0.04 | 0.02 | 0.29 |
|  |  |  |  |  |  | L, Ba | Colds | 12 | Boil leaves or bark and drink decoction; Eat ripe fruit |  |  |  |  |
|  |  |  |  |  |  | L, Ba | Cough | 12 | Boil leaves or bark and drink decoction |  |  |  |  |
|  |  |  |  |  |  | L, Ba | Indigestion | 13 | Boil leaves or bark and drink decoction; Eat ripe fruit |  |  |  |  |
|  |  |  |  |  |  | Ba | Eczema | 14 | Boil bark and soak affected part in the decoction; Pound bark into a paste and apply as poultice |  |  |  |  |
|  |  |  |  |  |  | L, Ba | Fever | 21 | Boil leaves or bark and drink decoction; Eat ripe fruit |  |  |  |  |
| Annonaceae | *Annona muricata* L. | Soursop (Eng.), Guyabano (Tag.), Bayubana (Ilk.) | CNu | LC | T | L | Dysentery | 1 | Boil leaves and drink decoction | 81 | 0.32 | 0.19 | 0.58 |
|  |  |  |  |  |  | L | Flu | 1 | Boil leaves and drink decoction |  |  |  |  |
|  |  |  |  |  |  | L | Cancer | 2 | Boil leaves and drink decoction |  |  |  |  |
|  |  |  |  |  |  | L, Fr | Diabetes | 5 | Boil leaves and drink decoction; Eat ripe fruit |  |  |  |  |
|  |  |  |  |  |  | L | High Uric Acid | 5 | Boil leaves and drink decoction |  |  |  |  |
|  |  |  |  |  |  | L, Fr | Hypertension | 11 | Boil leaves and drink decoction; Eat ripe fruit |  |  |  |  |
|  |  |  |  |  |  | L | Cough | 12 | Boil leaves and drink decoction |  |  |  |  |
|  |  |  |  |  |  | L | Hyperacidity | 13 | Boil leaves and drink decoction |  |  |  |  |
|  |  |  |  |  |  | L | Inflamed/Swollen Muscles | 15 | Pound leaves into a paste and apply as poultice; Boil leaves and soak swollen muscles in the decoction |  |  |  |  |
|  |  |  |  |  |  | L | Muscle Pain | 15 | Pound leaves into a paste and apply as poultice; Boil leaves and soak painful muscles in the decoction |  |  |  |  |
|  |  |  |  |  |  | L | Irregular Menstruation | 16 | Boil leaves and drink decoction |  |  |  |  |
|  |  |  |  |  |  | L | Kidney Problems | 16 | Boil leaves and drink decoction |  |  |  |  |
|  |  |  |  |  |  | L | UTI | 16 | Boil leaves and drink decoction |  |  |  |  |
| Annonaceae | *Annona squamosa* L. | Sugar Apple (Eng.), Atis (Tag., Ilk.) | Nu | LC | T | L, Fr | Diabetes | 5 | Boil leaves and drink decoction; Eat ripe fruit | 15 | 0.06 | 0.04 | 0.36 |
|  |  |  |  |  |  | L, Fr | Hypertension | 11 | Boil leaves and drink decoction; Eat ripe fruit |  |  |  |  |
|  |  |  |  |  |  | L | Diarrhea | 13 | Boil leaves and drink decoction |  |  |  |  |
|  |  |  |  |  |  | L | UTI | 16 | Boil leaves and drink decoction |  |  |  |  |
|  |  |  |  |  |  | L | Fainting | 21 | Crush fresh leaves to release the oil and inhale the scent |  |  |  |  |
|  |  |  |  |  |  | L | *Nakablaawan* | 00 | Boil leaves and drink decoction |  |  |  |  |
| Apiaceae | *Centella asiatica* (L.) Urb. | Gotu kola (Eng.), Takip-kohol (Tag.), Laplapayag (Ilk.) | N | LC | H | L | Boils | 1 | Pound leaves into a paste and apply as poultice | 3 | 0.01 | 0.00 | 0.17 |
|  |  |  |  |  |  | L | Fever | 21 | Boil leaves and drink decoction |  |  |  |  |
|  |  |  |  |  |  | L | wounds | 22 | Pound leaves into a paste and apply as poultice |  |  |  |  |
| Apocynaceae | *Alstonia scholaris* (L.) R.Br. | Scholar's Tree (Eng.), Dita (Tag.), Alipauen (Ilk.) | N | LC | T | Ba | Malaria | 1 | Boil bark and drink decoction | 6 | 0.02 | 0.01 | 0.23 |
|  |  |  |  |  |  | Ba | Edema | 11 | Boil bark and drink decoction; Pound bark into a paste and apply as poultice |  |  |  |  |
|  |  |  |  |  |  | Ba | Diarrhea | 13 | Boil bark and drink decoction |  |  |  |  |
|  |  |  |  |  |  | Ba | Fever | 21 | Boil bark and drink decoction |  |  |  |  |
| Apocynaceae | *Catharanthus roseus* (L.) G.Don | Pink periwinkle (Eng.), Tsitsirika (Tag., Ilk.) | Nu | NL | H | L | Beri-beri | 5 | Boil leaves and drink decoction; Crush leaves to extract juice and drink | 11 | 0.04 | 0.03 | 0.24 |
|  |  |  |  |  |  | R | Irregular menstruation | 16 | Boil leaves and drink decoction |  |  |  |  |
|  |  |  |  |  |  | L | Stomachache | 13 | Pound leaves into a paste and apply as poultice; Boil leaves and wash skin ulcer with the decoction |  |  |  |  |
|  |  |  |  |  |  | L | Skin ulcer | 14 | Boil roots and drink decoction |  |  |  |  |
| Apocynaceae | *Tabernaemontana pandacaqui* Poir. | Banana bush (Eng.), Kampupot (Tag.), Kuribetbet (Ilk.) | N | LC | S | L | Wounds | 22 | Pound leaves into a paste and apply as poultice | 1 | 0.00 | 0.00 | 0.06 |
| Araceae | *Colocasia esculenta* (L.) Schott | Taro (Eng.), Gabi (Tag.), Aba (Ilk.) | N | LC | H | R | Diabetes | 5 | Boil root crop and eat. | 3 | 0.01 | 0.01 | 0.12 |
|  |  |  |  |  |  | L | Constipation | 13 | Boil leaves or cook for food and eat. |  |  |  |  |
| Arecaceae | *Areca catechu* L. | Areca Nut Palm (Eng.), Buñga (Tag.), Boa (Ilk.) | N | LC | T | Fr | Amoebiasis | 1 | Boil nuts and drink decoction | 10 | 0.04 | 0.02 | 0.18 |
|  |  |  |  |  |  | Fr | Intestinal worms | 1 | Boil nuts and drink decoction |  |  |  |  |
|  |  |  |  |  |  | F | Relapsing fever | 1 | Boil flower and drink decoction |  |  |  |  |
|  |  |  |  |  |  | Fr | Weak teeth | 13 | Chew nuts with *P. betle* leaf and *apog* or lime |  |  |  |  |
|  |  |  |  |  |  | Fr | Abdominal pain | 21 | Chew nuts with *P. betle* leaf and *apog* or lime |  |  |  |  |
| Arecaceae | *Cocos nucifera* L. | Coconut (Eng.), Niyog (Tag., Ilk.) | N | NL | T | Fr | Dry Hair | 14 | Extract oil from fruit and massage onto the scalp and hair | 8 | 0.03 | 0.01 | 0.18 |
|  |  |  |  |  |  | Fr | Dry Skin | 14 | Extract oil from fruit and massage onto the skin |  |  |  |  |
|  |  |  |  |  |  | Fr | UTI | 16 | Drink coconut juice |  |  |  |  |
|  |  |  |  |  |  | Fr | *Pasma* | 00 | Extract oil from fruit, warm and apply to affected part; Drink coconut juice |  |  |  |  |
| Asphodelaceae | *Aloe vera* L. | Aloe vera (Eng.), Sabila (Tag., Ilk.) | CN | NL | H | L | Dandruff | 14 | Massage extracted leaf gel onto the scalp | 11 | 0.04 | 0.02 | 0.13 |
|  |  |  |  |  |  | L | Dry hair | 14 | Massage extracted leaf gel onto the scalp and hair |  |  |  |  |
|  |  |  |  |  |  | L | Dry skin | 14 | Massage extracted leaf gel onto the skin |  |  |  |  |
|  |  |  |  |  |  | L | Hair fall | 14 | Massage extracted leaf gel onto the scalp |  |  |  |  |
|  |  |  |  |  |  | L | Bruises | 22 | Massage extracted leaf gel onto bruises |  |  |  |  |
|  |  |  |  |  |  | L | Wounds | 22 | Massage extracted leaf gel onto wounds |  |  |  |  |
| Asteraceae | *Artemisia indica* Willd. | Mugwort (Eng.), Damong Maria (Tag.), Erbaka (Ilk.) | Nu | NL | H | L | Skin diseases | 14 | Pound leaves into a paste and apply as poultice | 10 | 0.04 | 0.03 | 0.24 |
|  |  |  |  |  |  | L | Increase menstrual flow | 16 | Boil leaves and drink or use decoction for a warm bath |  |  |  |  |
|  |  |  |  |  |  | L | Irregular menstruation | 16 | Boil leaves and drink or use decoction for a warm bath |  |  |  |  |
|  |  |  |  |  |  | L | Post-partum care | 18 | Boil leaves and drink or use decoction for a warm bath |  |  |  |  |
|  |  |  |  |  |  | L | Wounds | 22 | Pound leaves into a paste and apply as poultice |  |  |  |  |
| Asteraceae | *Blumea balsamifera* (L.) DC. | Buffalo ears (Eng.), Sambong (Tag.), Subusub (Ilk.) | N | LC | S | L | Flu | 1 | Boil leaves and drink or use decoction for a warm or steam bath (*suob*). Can be used together with *V. arvensis*, *P. guajava* & *C. citratus* | 223 | 0.88 | 0.26 | 0.52 |
|  |  |  |  |  |  | L | Colds | 12 | Boil leaves and drink or use decoction for a warm or steam bath (*suob*). Can be used together with *V. arvensis,* *P. guajava* & *C. citratus* |  |  |  |  |
|  |  |  |  |  |  | L | Cough | 12 | Boil leaves and drink or use decoction for a warm or steam bath (*suob*). Can be used together with *V. arvensis*, *P. guajava* & *C. citratus* |  |  |  |  |
|  |  |  |  |  |  | L | Diarrhea | 13 | Boil leaves and drink decoction |  |  |  |  |
|  |  |  |  |  |  | L | Difficulty in Urinating | 16 | Boil leaves and drink decoction |  |  |  |  |
|  |  |  |  |  |  | L | Kidney Problems | 16 | Boil leaves and drink decoction |  |  |  |  |
|  |  |  |  |  |  | L | Kidney Stones | 16 | Boil leaves and drink decoction |  |  |  |  |
|  |  |  |  |  |  | L | Dysmenorrhea | 16 | Boil leaves and drink decoction |  |  |  |  |
|  |  |  |  |  |  | L | UTI | 16 | Boil leaves and drink decoction |  |  |  |  |
|  |  |  |  |  |  | L | Fever | 21 | Boil leaves and drink or use decoction for a warm or steam bath (*suob*). Can be used together with *V. arvensis*, *P. guajava* & *C. citratus* |  |  |  |  |
|  |  |  |  |  |  | L | Headache | 21 | Boil leaves and drink or use decoction for a warm or steam bath (*suob*). Can be used together with *V. arvensis*, *P. guajava* & *C. citratus* |  |  |  |  |
|  |  |  |  |  |  | L | *Pasma* | 00 | Boil leaves and drink or use decoction for a warm or steam bath (*suob*). Can be used together with *V. arvensis*, *P. guajava* & *C. citratus* |  |  |  |  |
| Asteraceae | *Chromolaena odorata* (L.) R.M.King & H.Rob. | Bitter bush (Eng.), Hagonoy (Tag.), Pantaleon (Ilk.) | Nu | NL | H | L | Wounds | 22 | Pound leaves into a paste and apply as poultice | 3 | 0.01 | 0.01 | 0.06 |
| Asteraceae | *Cyanthillium cinereum* (L.) H.Rob. | Iron weed (Eng.), Tagulinay (Tag.), Agas-moro (Ilk.) | N | NL | H | L | Cough | 12 | Boil leaves and drink decoction | 2 | 0.01 | 0.00 | 0.11 |
|  |  |  |  |  |  | L | Wounds | 22 | Pound leaves into a paste and apply as poultice |  |  |  |  |
| Bignoniaceae | *Crescentia cujete* L. | Calabash Tree (Eng.), Kalabas (Tag.), Miracle fruit (Ilk.) | CNu | LC | T | L, Fr | Hypertension | 11 | Boil leaves or fruit pulp and drink decoction | 9 | 0.04 | 0.01 | 0.18 |
|  |  |  |  |  |  | L, Fr | Asthma | 12 | Boil leaves or fruit pulp and drink decoction |  |  |  |  |
|  |  |  |  |  |  | L, Fr | Cough | 12 | Boil leaves or fruit pulp and drink decoction |  |  |  |  |
|  |  |  |  |  |  | Ba | Diarrhea | 13 | Boil leaves or fruit pulp and drink decoction |  |  |  |  |
|  |  |  |  |  |  | L, Fr | Stomachache | 13 | Boil leaves or fruit pulp and drink decoction |  |  |  |  |
| Bixaceae | *Bixa orellana* L. | Lipstick plant (Eng.), Atsuete (Tag.), Atsuite (Ilk.) | Nu | LC | T | L | Fever | 21 | Boil leaves and drink decoction | 5 | 0.02 | 0.01 | 0.12 |
|  |  |  |  |  |  | L, Se | Burns | 22 | Pound leaves or seeds into a paste and apply as poultice |  |  |  |  |
|  |  |  |  |  |  | L | Wounds | 22 | Pound leaves into a paste and apply as poultice |  |  |  |  |
| Bombacaceae | *Ceiba pentandra* (L.) Gaertn. | Cottonwood (Eng.), Kapok (Tag.), Kapas-sanglai (Ilk.) | Nu | LC | T | Ba | Asthma | 12 | Boil bark and drink decoction | 6 | 0.02 | 0.02 | 0.18 |
|  |  |  |  |  |  | Ba | Cough | 12 | Boil bark and drink decoction |  |  |  |  |
|  |  |  |  |  |  | Ba | Diarrhea | 13 | Boil bark and drink decoction |  |  |  |  |
|  |  |  |  |  |  | Ba | Sprain | 22 | Pound bark into a paste and apply as poultice |  |  |  |  |
| Calophyllaceae | *Calophyllum inophyllum* L. | Ball Nut Tree (Eng.), Palo Maria (Tag.), Bitaog (Ilk.) | N | LC | T | Se | Dysentery | 1 | Boil seeds and drink decoction | 12 | 0.05 | 0.04 | 0.19 |
|  |  |  |  |  |  | L | Eye Infection | 9 | Boil leaves and wash eyes with the decoction |  |  |  |  |
|  |  |  |  |  |  | R | Gas Pain | 13 | Boil roots and drink decoction |  |  |  |  |
| Caricaceae | *Carica papaya* L. | Papaya (Eng., Tag., Ilk.) | Nu | DD | T | L | Dengue Fever | 1 | Boil leaves and drink decoction; Crush leaves to extract juice and drink | 16 | 0.06 | 0.04 | 0.14 |
|  |  |  |  |  |  | Fr | Constipation | 13 | Eat ripe fruit |  |  |  |  |
|  |  |  |  |  |  | L, Fr | Fever | 21 | Boil leaves and drink decoction; Eat ripe fruit |  |  |  |  |
| Commelinaceae | *Commelina benghalensis* L. | Benghal Day Flower (Eng.), Bias-bias (Tag.), Kulkul-lasi (Ilk.) | N | LC | H | L | Boils | 1 | Pound leaves into a paste and apply as poultice | 7 | 0.03 | 0.02 | 0.18 |
|  |  |  |  |  |  | L | Warts | 1 | Pound leaves into a paste and apply as poultice |  |  |  |  |
|  |  |  |  |  |  | L | Abscess | 1 | Pound leaves into a paste and apply as poultice |  |  |  |  |
|  |  |  |  |  |  | L | Eczema | 14 | Pound leaves into a paste and apply as poultice |  |  |  |  |
|  |  |  |  |  |  | L | Wounds | 22 | Pound leaves into a paste and apply as poultice |  |  |  |  |
| Convolvulaceae | *Ipomoea aquatica* Forssk. | Water Spinach (Eng.), Kangkong (Tag., Ilk.) | N | LC | V | L | Scabies | 1 | Boil leaves and wash scabies with the decoction; Crush leaves to extract juice and apply it to the scabies | 7 | 0.03 | 0.02 | 0.18 |
|  |  |  |  |  |  | L | Ringworm | 1 | Boil leaves and wash ringworms with the decoction; Crush leaves to extract juice and apply it to the ringworms |  |  |  |  |
|  |  |  |  |  |  | L | Hypertension | 11 | Eat cooked leaves |  |  |  |  |
|  |  |  |  |  |  | L | Constipation | 13 | Boil leaves and drink decoction |  |  |  |  |
| Convolvulaceae | *Ipomoea batatas* (L.) Lam. | Sweet potato (Eng.), Kamote (Tag., Ilk.) | CNu | DD | V | L, R | Diabetes | 5 | Eat boiled or cooked leaves and root crop. | 2 | 0.01 | 0.00 | 0.11 |
|  |  |  |  |  |  | L, R | Constipation | 13 | Eat boiled or cooked leaves and root crop. |  |  |  |  |
| Cordiaceae | *Cordia dichotoma* G.Forst. | Soap berry (Eng.), Anonang (Tag., Ilk.) | N | LC | T | L | Headache | 21 | Boil leaves and drink decoction | 1 | 0.00 | 0.00 | 0.06 |
| Cucurbitaceae | *Cucumis sativus* L. | Cucumber (Eng.), Pipino (Tag., Ilk.) | CNu | NL | V | Fr | High Cholesterol | 5 | Eat fruit | 17 | 0.07 | 0.06 | 0.26 |
|  |  |  |  |  |  | Fr | Dry skin | 14 | Extract fruit juice using blender and apply to rough skin |  |  |  |  |
|  |  |  |  |  |  | Fr | Puffy eyes | 9 | Wash, slice fruit and place directly over closed eyes |  |  |  |  |
|  |  |  |  |  |  | Fr | Scalds | 22 | Extract fruit juice using blender and apply to scalds |  |  |  |  |
|  |  |  |  |  |  | Fr | Sunburn | 22 | Extract fruit juice using blender and apply to sunburns |  |  |  |  |
| Cucurbitaceae | *Momordica charantia* L. | Bitter gourd (Eng.), Ampalaya (Tag.), Parya (Ilk.) | Nu | NL | V | L, Fr | Diabetes | 5 | Extract fruit juice using blender and drink; Eat cooked fruit and leaves | 6 | 0.02 | 0.02 | 0.18 |
|  |  |  |  |  |  | Fr | Hives | 14 | Pound fruit into a paste and apply as poultice |  |  |  |  |
|  |  |  |  |  |  | L | Fever | 21 | Boil leaves and drink decoction |  |  |  |  |
| Euphorbiaceae | *Euphorbia hirta* L. | Snake weed (Eng.), Tawa-tawa (Tag., Ilk.) | Nu | NL | H | L | Dengue fever | 1 | Boil leaves and drink decoction | 34 | 0.13 | 0.10 | 0.24 |
|  |  |  |  |  |  | L | Flu | 1 | Boil leaves and drink decoction |  |  |  |  |
|  |  |  |  |  |  | L | Hypertension | 11 | Boil leaves and drink decoction |  |  |  |  |
|  |  |  |  |  |  | L | Fever | 21 | Boil leaves and drink decoction |  |  |  |  |
| Euphorbiaceae | *Jatropha curcas* L. | Physic Nut Tree (Eng.), Tubang bakod (Tag.), Tagumbao (Ilk.) | CNu | LC | S | L | Toothache | 13 | Pound leaves into a paste and apply as poultice | 7 | 0.03 | 0.01 | 0.18 |
|  |  |  |  |  |  | L | Diarrhea | 13 | Boil leaves and drink decoction |  |  |  |  |
|  |  |  |  |  |  | L | Fever | 21 | Boil leaves and drink decoction |  |  |  |  |
|  |  |  |  |  |  | L | Snake bite | 22 | Pound leaves into a paste and apply as poultice |  |  |  |  |
|  |  |  |  |  |  | L | Wounds | 22 | Pound leaves into a paste and apply as poultice |  |  |  |  |
| Fabaceae | *Abrus precatorius* L. | Prayer beads (Eng.), Saga (Tag.), Bugbugayong (Ilk.) | N | NL | V | R | Cough | 12 | Boil roots and drink decoction | 2 | 0.01 | 0.00 | 0.11 |
|  |  |  |  |  |  | R | Fever | 21 | Boil roots and drink decoction |  |  |  |  |
| Fabaceae | *Cassia fistula* L. | Golden Shower (Eng., Ilk.), Palucheba (Tag.) | Nu | LC | T | L, F | Constipation | 13 | Boil leaves or flowers and drink decoction | 1 | 0.00 | 0.00 | 0.06 |
| Fabaceae | *Clitoria ternatea* L. | Blue pea (Eng.), Pukingan (Tag.), Samsamping (Ilk.) | Nu | NL | V | L, F | Boils | 1 | Pound leaves or flowers into a paste and apply as poultice | 18 | 0.07 | 0.06 | 0.21 |
|  |  |  |  |  |  | L, F | Rheumatism | 15 | Boil leaves or flowers and drink decoction; Pound leaves or flowers into a paste and apply as poultice |  |  |  |  |
|  |  |  |  |  |  | L, F | Wounds | 22 | Pound leaves or flowers into a paste and apply as poultice |  |  |  |  |
| Fabaceae | *Gliricidia sepium* (Jacq.) Kunth | Mother of cocoa (Eng.), Kakawate (Tag., Ilk.) | Nu | LC | T | L | Scabies | 1 | Pound and crush leaves to make a paste. Apply paste directly to scabies as poultice; Boil leaves to make a decoction. Use decoction to wash infected part. | 2 | 0.01 | 0.00 | 0.11 |
|  |  |  |  |  |  | L, Ba | Wounds | 22 | Pound and crush leaves to make a paste. Apply paste directly to wounds as poultice; Boil leaves and bark to make a decoction. Use decoction to wash wounds. |  |  |  |  |
| Fabaceae | *Leucaena leucocephala*  (Lam.) de Wit | Lead Tree (Eng.), Ipil-ipil (Tag., Ilk.) | Nu | NL | T | L | Malaria | 1 | Boil leaves and drink decoction | 7 | 0.03 | 0.02 | 0.18 |
|  |  |  |  |  |  | L | Dysentery | 1 | Boil leaves and drink decoction |  |  |  |  |
|  |  |  |  |  |  | L | Diarrhea | 13 | Boil leaves and drink decoction |  |  |  |  |
|  |  |  |  |  |  | L | Fever | 21 | Boil leaves and drink decoction |  |  |  |  |
| Fabaceae | *Mimosa pudica* L. | Bashful mimosa (Eng.), Makahiya (Tag.), Bain-bain (Ilk.) | Nu | LC | H | L | Mumps | 1 | Pound leaves into a paste and apply as poultice | 14 | 0.06 | 0.03 | 0.30 |
|  |  |  |  |  |  | L | Diabetes | 5 | Boil leaves and drink decoction |  |  |  |  |
|  |  |  |  |  |  | L | Hypertension | 11 | Boil leaves and drink decoction |  |  |  |  |
|  |  |  |  |  |  | L | Asthma | 12 | Boil leaves and drink decoction |  |  |  |  |
|  |  |  |  |  |  | L | Cough | 12 | Boil leaves and drink decoction |  |  |  |  |
|  |  |  |  |  |  | L | Dysmenorrhea | 16 | Boil leaves and drink decoction |  |  |  |  |
|  |  |  |  |  |  | L | Kidney problems | 16 | Boil leaves and drink decoction |  |  |  |  |
|  |  |  |  |  |  | L | Kidney stones | 16 | Boil leaves and drink decoction |  |  |  |  |
|  |  |  |  |  |  | L | UTI | 16 | Boil leaves and drink decoction |  |  |  |  |
| Fabaceae | *Pithecellobium dulce* (Roxb.) Benth. | Sweet Tamarind (Eng.), Kamatsile (Tag.), Damortis (Ilk.) | Nu | LC | T | Ba | Dysentery | 1 | Boil bark and drink decoction | 6 | 0.02 | 0.01 | 0.23 |
|  |  |  |  |  |  | Fr | Diabetes | 5 | Eat fruit |  |  |  |  |
|  |  |  |  |  |  | Ba | Indigestion | 13 | Boil bark and drink decoction |  |  |  |  |
|  |  |  |  |  |  | L, Ba | Wounds | 22 | Boil leaves and drink decoction; Pound bark into a paste and apply as poultice |  |  |  |  |
| Fabaceae | *Psophocarpus tetragonolobus* (L.) DC. | Winged Beans (Eng.), Sigarilyas (Tag., Ilk.) | Nu | NL | V | Fr | Diabetes | 5 | Cook and eat fruit | 5 | 0.02 | 0.02 | 0.13 |
|  |  |  |  |  |  | Fr | High Cholesterol | 5 | Cook and eat fruit |  |  |  |  |
|  |  |  |  |  |  | Fr | Irregular Bowel Movement | 13 | Cook and eat fruit |  |  |  |  |
| Fabaceae | *Samanea saman* (Jacq.) Merr. | Rain Tree (Eng.), Akasya (Tag., Ilk.) | Nu | LC | T | L, Ba | Dysentery | 1 | Boil leaves or bark and drink decoction | 15 | 0.06 | 0.04 | 0.19 |
|  |  |  |  |  |  | L, Ba | Diarrhea | 13 | Boil leaves or bark and drink decoction |  |  |  |  |
|  |  |  |  |  |  | L, Ba | Stomachache | 13 | Boil leaves or bark and drink decoction |  |  |  |  |
|  |  |  |  |  |  | L | Wounds | 22 | Pound leaves into a paste and apply as poultice |  |  |  |  |
| Fabaceae | *Senna alata* (L.) Roxb. | Candelabra bush (Eng.), Akapulko (Tag.), Andadasi (Ilk.) | Nu | LC | S | L | Cough | 12 | Boil leaves and drink decoction | 3 | 0.01 | 0.00 | 0.17 |
|  |  |  |  |  |  | L | Skin infection | 14 | Pound leaves into a paste and apply as poultice |  |  |  |  |
|  |  |  |  |  |  | L | Insect bite | 22 | Pound leaves into a paste and apply as poultice |  |  |  |  |
| Fabaceae | *Tamarindus indica* L. | Tamarind (Eng.), Sampalok (Tag.), Salamagi (Ilk.) | Nu | LC | T | L, Fr | Flu | 1 | Boil leaves in water to make a decoction. Drink 1 glass/cup of decoction 2-3 times a day; Eat ripe fruit. | 12 | 0.05 | 0.04 | 0.31 |
|  |  |  |  |  |  | L | Sore eyes | 9 | Boil young leaves in a small amount of water. Cool, crush, and strain the leaves to extract the juice. Put 1-2 drops of juice in affected eyes. |  |  |  |  |
|  |  |  |  |  |  | L, Fr | Asthma | 12 | Boil leaves in water to make a decoction. Drink 1 glass/cup of decoction 2-3 times a day; Eat ripe fruit. |  |  |  |  |
|  |  |  |  |  |  | L, Fr | Cough | 12 | Boil leaves in water to make a decoction. Z. officinale can be added. Drink 1 glass/cup of decoction 2-3 times a day ; Eat ripe fruit. |  |  |  |  |
|  |  |  |  |  |  | L, Fr | Diarrhea | 13 | Boil leaves in water to make a decoction. Drink 1 glass/cup of decoction 2-3 times a day; Eat ripe fruit. |  |  |  |  |
|  |  |  |  |  |  | L, Fr | Fever | 21 | Boil leaves in water to make a decoction. Drink 1 glass/cup of decoction 2-3 times a day; Eat ripe fruit. |  |  |  |  |
| Lamiaceae | *Coleus amboinicus* Lour. | Oregano (Eng., Ilk.), Suganda (Tag.) | CNu | NL | H | L | Flu | 1 | Boil leaves and drink decoction | 166 | 0.66 | 0.49 | 0.62 |
|  |  |  |  |  |  | L | Asthma | 12 | Crush leaves to extract the juice and drink; Infuse leaves in hot water and drink infusion |  |  |  |  |
|  |  |  |  |  |  | L | Bronchitis | 12 | Crush leaves to extract the juice and drink; Infuse leaves in hot water and drink infusion |  |  |  |  |
|  |  |  |  |  |  | L | Cough | 12 | Crush leaves to extract the juice and drink; Infuse leaves in hot water and drink infusion |  |  |  |  |
|  |  |  |  |  |  | L | Sore throat | 12 | Crush leaves to extract the juice and drink; Infuse leaves in hot water and drink infusion |  |  |  |  |
|  |  |  |  |  |  | L | Inflamed/Swollen Muscles | 15 | Pound leaves into a paste and apply as poultice |  |  |  |  |
|  |  |  |  |  |  | L | Fever | 21 | Boil leaves and drink decoction |  |  |  |  |
|  |  |  |  |  |  | L | Headache | 21 | Boil leaves and drink decoction |  |  |  |  |
|  |  |  |  |  |  | L | Burns | 22 | Pound leaves into a paste and apply as poultice; Boil leaves and wash burns with the decoction |  |  |  |  |
|  |  |  |  |  |  | L | Wounds | 22 | Pound leaves into a paste and apply as poultice; Boil leaves and wash wounds with the decoction |  |  |  |  |
| Lamiaceae | *Coleus scutellarioides* (L.) Benth. | Coleus plant (Eng.), Mayana (Tag.), Dara-dara (Ilk.) | N | NL | H | L | Boils | 1 | Pound leaves into a paste and apply as poultice | 9 | 0.04 | 0.02 | 0.18 |
|  |  |  |  |  |  | L | Headache | 21 | Pound leaves into a paste and apply as poultice; Boil leaves and drink or use decoction for a warm bath or steam bath (*suob*) |  |  |  |  |
|  |  |  |  |  |  | L | Bruises | 22 | Pound leaves into a paste and apply as poultice |  |  |  |  |
|  |  |  |  |  |  | L | Wounds | 22 | Pound leaves into a paste and apply as poultice |  |  |  |  |
| Lamiaceae | *Gmelina philippensis* Cham. | Gmelina (Eng.), Alipung (Tag.), Bosel-bosel (Ilk.) | N | LC | T | L, Ba | Flu | 1 | Boil leaves or bark and drink decoction | 2 | 0.01 | 0.00 | 0.11 |
|  |  |  |  |  |  | L, Ba | Fever | 21 | Boil leaves or bark and drink decoction |  |  |  |  |
| Lamiaceae | *Ocimum tenuiflorum* L. | Holy basil (Eng.), Sulasi (Tag.), Biday (Ilk.) | N | NL | H | L | Allergies | 4 | Boil leaves and drink decoction; Pound leaves into a paste and apply as poultice | 10 | 0.04 | 0.02 | 0.35 |
|  |  |  |  |  |  | L | Hives | 14 | Boil leaves and drink decoction; Pound leaves into a paste and apply as poultice |  |  |  |  |
|  |  |  |  |  |  | L | Hypertension | 11 | Boil leaves and drink decoction |  |  |  |  |
|  |  |  |  |  |  | L | Cough | 12 | Boil leaves and drink decoction |  |  |  |  |
|  |  |  |  |  |  | L | Gas pain | 13 | Boil leaves and drink decoction |  |  |  |  |
|  |  |  |  |  |  | L | Toothache | 13 | Pound leaves into a paste and apply as poultice |  |  |  |  |
|  |  |  |  |  |  | L | Muscle cramps | 15 | Boil leaves and drink decoction |  |  |  |  |
|  |  |  |  |  |  | L | Wounds | 22 | Pound leaves into a paste and apply as poultice |  |  |  |  |
| Lamiaceae | *Orthosiphon aristatus* (Blume) Miq. | Cat's Whiskers (Eng.), Balbas-pusa (Tag.), Tahibo (Ilk.) | N | NL | H | L | Diuretic | 11 | Boil leaves and drink decoction | 4 | 0.02 | 0.01 | 0.17 |
|  |  |  |  |  |  | L | Gout | 15 | Boil leaves and drink decoction |  |  |  |  |
|  |  |  |  |  |  | L | UTI | 16 | Boil leaves and drink decoction |  |  |  |  |
| Lamiaceae | *Premna odorata* Blanco | Fragrant premna (Eng.), Alagaw (Tag., Ilk.) | N | LC | T | L | Cough | 12 | Boil leaves and drink decoction | 1 | 0.00 | 0.00 | 0.06 |
| Lamiaceae | *Vitex arvensis* Gentallan, Sengun & M.B. Bartolome | Five-leaved Chaste Tree (Eng.), Lagundi (Tag.), Dangla (Ilk.) | N, E | LC | S | L | Flu | 1 | Boil leaves and drink or use decoction for a warm or steam bath (*suob*). Can be used together with *B. balsamifera*, *P. guajava* & *C. citratus* | 387 | 1.54 | 0.71 | 1.00 |
|  |  |  |  |  |  | L | Relapsing fever | 1 | Boil leaves and drink or use decoction for a warm or steam bath (*suob*). Can be used together with *B. balsamifera*, *P. guajava* & *C. citratus* |  |  |  |  |
|  |  |  |  |  |  | L | Hypertension | 11 | Boil leaves and drink decoction |  |  |  |  |
|  |  |  |  |  |  | L | Asthma | 12 | Boil leaves and drink decoction |  |  |  |  |
|  |  |  |  |  |  | L | Cough | 12 | Boil leaves and drink decoction |  |  |  |  |
|  |  |  |  |  |  | L | Sore throat | 12 | Boil leaves and drink decoction |  |  |  |  |
|  |  |  |  |  |  | L | Diarrhea | 13 | Boil leaves and drink decoction |  |  |  |  |
|  |  |  |  |  |  | L | Stomachache | 13 | Boil leaves and drink decoction |  |  |  |  |
|  |  |  |  |  |  | L | Rheumatism | 15 | Boil leaves and drink decoction; Pound leaves into a paste and apply as poultice |  |  |  |  |
|  |  |  |  |  |  | L | Post-Partum care | 18 | Boil leaves and use decoction for bathing |  |  |  |  |
|  |  |  |  |  |  | L | Fever | 21 | Boil leaves and drink or use decoction for a warm or steam bath (*suob*). Can be used together with *B. balsamifera*, *P. guajava* & *C. citratus* |  |  |  |  |
|  |  |  |  |  |  | L | Headache | 21 | Boil leaves and drink decoction |  |  |  |  |
|  |  |  |  |  |  | L | Wounds | 22 | Pound leaves into a paste and apply as poultice; Boil leaves and use decoction for bathing |  |  |  |  |
|  |  |  |  |  |  | L | Pasma | 00 | Boil leaves and use decoction for a warm bath |  |  |  |  |
| Lauraceae | *Persea americana* Mill. | Avocado (Eng.), Abukado (Tag., Ilk.) | CN | LC | T | L, Ba | Dysentery | 1 | Boil leaves or bark and drink decoction | 6 | 0.02 | 0.01 | 0.18 |
|  |  |  |  |  |  | L | Diabetes | 5 | Boil leaves and drink decoction |  |  |  |  |
|  |  |  |  |  |  | L, Se | Diarrhea | 13 | Boil leaves or dried seeds and drink decoction |  |  |  |  |
| Lythraceae | *Lagerstroemia speciosa* (L.) Pers. | Queen of Flowers (Eng.), Banaba (Tag., Ilk.) | N | LC | T | L | Flu | 1 | Boil leaves and drink or use decoction for a warm bath or steam bath (*suob*). Can be used together with *V. arvensis*, *B. balsamifera*, *C. citratus*, and *P. guajava* | 51 | 0.20 | 0.10 | 0.35 |
|  |  |  |  |  |  | L | Diabetes | 5 | Boil and drink decoction |  |  |  |  |
|  |  |  |  |  |  | L | High Cholesterol | 5 | Boil and drink decoction |  |  |  |  |
|  |  |  |  |  |  | L | Hypertension | 11 | Boil and drink decoction |  |  |  |  |
|  |  |  |  |  |  | L | Kidney Problems | 16 | Boil and drink decoction |  |  |  |  |
|  |  |  |  |  |  | L | Kidney Stones | 16 | Boil and drink decoction |  |  |  |  |
|  |  |  |  |  |  | L | UTI | 16 | Boil and drink decoction |  |  |  |  |
|  |  |  |  |  |  | L | Fever | 21 | Boil leaves and drink or use decoction for a warm bath or steam bath (*suob*). Can be used together with *V. arvensis*, *B. balsamifera*, *C. citratus*, and *P. guajava* |  |  |  |  |
| Malvaceae | *Abelmoschus esculentus* (L.) Moench | Ladie's Finger (Eng.), Okra (Tag., Ilk.) | CNu | NL | H | Fr | Diabetes | 5 | Infuse fruit in water overnight and drink infusion; Eat cooked fruit | 8 | 0.03 | 0.02 | 0.07 |
|  |  |  |  |  |  | Fr | High Cholesterol | 5 | Infuse fruit in water overnight and drink infusion; Eat cooked fruit |  |  |  |  |
| Malvaceae | *Hibiscus rosa-sinensis* L. | Hibiscus (Eng.), Gumamela (Tag., Ilk.) | Nu | NL | S | L, F | Boils | 1 | Pound and crush leaves and flower into a paste. Apply paste directly to boils as poultice. | 7 | 0.03 | 0.02 | 0.13 |
|  |  |  |  |  |  | L, F | Wounds | 22 | Pound and crush leaves and flower into a paste. Apply paste directly to boils as poultice. |  |  |  |  |
| Malvaceae | *Malvastrum coromandelianum* (L.) Garcke | False mallow (Eng.), Kinaylumpang (Tag.), Gagabuten (Ilk.) | Nu | NL | H | L | Hypertension | 11 | Boil leaves and drink decoction | 2 | 0.01 | 0.00 | 0.11 |
|  |  |  |  |  |  | L | UTI | 16 | Boil leaves and drink decoction |  |  |  |  |
| Meliaceae | *Azadirachta indica* A.Juss. | Neem Tree (Eng., Tag., Ilk.) | Nu | LC | T | L | Flu | 1 | Boil leaves and drink decoction | 2 | 0.01 | 0.00 | 0.11 |
|  |  |  |  |  |  | L | Fever | 21 | Boil leaves and drink decoction |  |  |  |  |
| Meliaceae | *Sandoricum koetjape* (Burm.f.) Merr. | Lolly Fruit (Eng.), Santol (Tag., Ilk.) | N | LC | T | L | Dysentery | 1 | Boil leaves and drink decoction | 12 | 0.05 | 0.04 | 0.19 |
|  |  |  |  |  |  | Ba | Indigestion | 13 | Boil bark and drink decoction |  |  |  |  |
|  |  |  |  |  |  | Ba | Diarrhea | 13 | Boil bark and drink decoction |  |  |  |  |
|  |  |  |  |  |  | L | Fever | 21 | Boil leaves and use decoction for a warm bath |  |  |  |  |
| Meliaceae | *Swietenia macrophylla* King | Mahogany (Eng., Tag., Ilk.) | Nu | EN | T | L | Hypertension | 11 | Boil leaves and drink decoction | 9 | 0.04 | 0.03 | 0.19 |
|  |  |  |  |  |  | Ba | Dysmenorrhea | 16 | Boil bark and drink decoction |  |  |  |  |
|  |  |  |  |  |  | Se | Abortifacient | 19 | Boil seeds and drink decoction |  |  |  |  |
| Moraceae | *Artocarpus heterophyllus* Lam. | Jackfruit (Eng.), Langka (Tag., Ilk.) | Nu | NL | T | L | Diabetes | 5 | Eat boiled seeds; Eat ripe fruit | 6 | 0.02 | 0.02 | 0.18 |
|  |  |  |  |  |  | L | Arthritis | 15 | Boil leaves and drink decoction |  |  |  |  |
|  |  |  |  |  |  | L | Wound | 22 | Pound leaves into a paste and apply as poultice |  |  |  |  |
| Moraceae | *Ficus septica Burn. f.* | Hauili Fig Tree (Eng.), Hauili (Tag.), Lapting (Ilk.) | N | NL | T | Fr | Wound | 22 | Extract fruit sap and apply topically to affected part | 1 | 0.00 | 0.00 | 0.06 |
| Moringaceae | *Moringa oleifera* Lam. | Horseradish Tree (Eng.), Malunggay (Tag.), Marunggay (Ilk.) | CNu | LC | T | L, Fr, Se | Anemia | 3 | Boil leaves, fruits, or seeds and drink decoction; Eat cooked leaves, fruits, or seeds | 21 | 0.08 | 0.05 | 0.37 |
|  |  |  |  |  |  | L, Fr, Se | Diabetes | 5 | Boil leaves, fruits, or seeds and drink decoction; Eat cooked leaves, fruits, or seeds |  |  |  |  |
|  |  |  |  |  |  | L, Fr, Se | Hypertension | 11 | Boil leaves, fruits, or seeds and drink decoction; Eat cooked leaves, fruits, or seeds |  |  |  |  |
|  |  |  |  |  |  | L, Fr, Se | Constipation | 13 | Boil leaves, fruits, or seeds and drink decoction; Eat cooked leaves, fruits, or seeds |  |  |  |  |
|  |  |  |  |  |  | L, Fr, Se | Rheumatism | 15 | Boil leaves, fruits, or seeds and drink decoction; Eat cooked leaves, fruits, or seeds |  |  |  |  |
|  |  |  |  |  |  | L, Fr, Se | Increase Breastmilk Production | 18 | Boil leaves, fruits, or seeds and drink decoction; Eat cooked leaves, fruits, or seeds |  |  |  |  |
| Musaceae | *Musa paradisiaca* L. | Banana (Eng.), Saging (Tag.), Saba (Ilk.) | N | NL | H | Fr | Diarrhea | 13 | Eat semi-ripe fruit. | 4 | 0.02 | 0.01 | 0.17 |
|  |  |  |  |  |  | Fr | Muscle cramps | 15 | Eat ripe fruit. |  |  |  |  |
|  |  |  |  |  |  | L | Fever | 21 | Heat leaves over hot surface. Apply leaves to forehead, back or chest. |  |  |  |  |
| Myrtaceae | *Psidium guajava* L. | Guava (Eng.) Bayabas (Tag., Ilk.) | Nu | LC | T | L | Boils | 1 | Pound leaves into a paste and apply as poultice; Boil leaves and wash boils with the decoction | 83 | 0.33 | 0.18 | 0.57 |
|  |  |  |  |  |  | L | Flu | 1 | Boil leaves and drink or use decoction for a warm bath or steam bath (**suob**). Can be used together with *V. negundo*, *B. balsamifera*, and *C. citratus* |  |  |  |  |
|  |  |  |  |  |  | L | Diabetes | 5 | Boil leaves and drink decoction |  |  |  |  |
|  |  |  |  |  |  | L | Hypertension | 11 | Boil leaves and drink decoction |  |  |  |  |
|  |  |  |  |  |  | L | Colds | 12 | Boil leaves and drink decoction |  |  |  |  |
|  |  |  |  |  |  | L | Cough | 12 | Boil leaves and drink decoction |  |  |  |  |
|  |  |  |  |  |  | L, Fr | Diarrhea | 13 | Boil leaves and drink decoction; Eat unripe fruit |  |  |  |  |
|  |  |  |  |  |  | L | Skin ulcer | 14 | Pound leaves into a paste and apply as poultice; Boil leaves and wash skin ulcers with the decoction |  |  |  |  |
|  |  |  |  |  |  | L | Fever | 21 | Boil leaves and drink or use decoction for a warm bath or steam bath (*suob*). Can be used together with *V. negundo*, *B. balsamifera*, and *C. citratus* |  |  |  |  |
|  |  |  |  |  |  | L | Bruises | 22 | Pound leaves into a paste and apply as poultice; Boil leaves and wash bruises with the decoction |  |  |  |  |
|  |  |  |  |  |  | L | Cuts | 22 | Pound leaves into a paste and apply as poultice; Boil leaves and wash cuts with the decoction |  |  |  |  |
|  |  |  |  |  |  | L | Wounds | 22 | Pound leaves into a paste and apply as poultice; Boil leaves and wash wounds with the decoction |  |  |  |  |
| Myrtaceae | *Syzygium cumini* L. | Black plum (Eng.), Duhat (Tag.), Lomboy (Ilk.) | Nu | LC | T | Ba | Tonsilitis | 12 | Boil bark and gargle with the decoction | 8 | 0.03 | 0.02 | 0.13 |
|  |  |  |  |  |  | Ba | Sore throat | 12 | Boil bark and gargle with the decoction |  |  |  |  |
|  |  |  |  |  |  | L | Diarrhea | 13 | Boil leaves and drink decoction |  |  |  |  |
| Oxalidaceae | *Averrhoa bilimbi* L. | Bilimbi Tree (Eng.), Kamias (Tag.), Pias (Ilk.) | Nu | LC | T | F | Varicose veins | 11 | Boil flowers and soak legs with varicose veins in the decoction | 6 | 0.02 | 0.02 | 0.18 |
|  |  |  |  |  |  | Fr | Constipation | 13 | Eat fruit |  |  |  |  |
|  |  |  |  |  |  | L, Fr | Vomiting | 13 | Boil leaves or fruit and drink decoction |  |  |  |  |
|  |  |  |  |  |  | Fr | Fever | 21 | Eat fruit; Extract fruit juice and drink |  |  |  |  |
| Pandanaceae | *Pandanus amaryllifolius*  Roxb. ex Lindl. | Fragrant Pandan (Eng.), Pandan (Tag., Ilk.) | N | DD | H | L | Diabetes | 5 | Boil leaves and drink decoction | 29 | 0.12 | 0.08 | 0.22 |
|  |  |  |  |  |  | L | High Cholesterol | 5 | Boil leaves and drink decoction |  |  |  |  |
|  |  |  |  |  |  | L | Hypertension | 11 | Boil leaves and drink decoction |  |  |  |  |
|  |  |  |  |  |  | L | Dandruff | 14 | Boil leaves and massage decoction onto the hair and scalp. Can be used with coconut oil. |  |  |  |  |
| Passifloraceae | *Passiflora foetida* L. | Bush passion fruit (Eng.), Marya-marya (Tag.), Kitkitiwit (Ilk.) | Nu | NL | V | L | Wounds | 22 | Boil leaves to make a decoction. Use decoction to wash wounds; Pound fruit into a paste and apply as poultice | 1 | 0.00 | 0.00 | 0.06 |
| Phyllanthaceae | *Phyllanthus acidus* (L.) Skeels | Gooseberry (Eng.), Iba (Tag.), Karmay (Ilk.) | Nu | NL | T | Fr | Hives | 14 | Pound fruit into a paste and apply as poultice | 7 | 0.03 | 0.02 | 0.23 |
|  |  |  |  |  |  | Fr | Rashes | 14 | Pound fruit into a paste and apply as poultice |  |  |  |  |
|  |  |  |  |  |  | Fr | Post partum care | 18 | Extract fruit juice using a blender and drink; Eat fruit |  |  |  |  |
|  |  |  |  |  |  | Fr | Insect bites | 22 | Pound fruit into a paste and apply as poultice |  |  |  |  |
| Piperaceae | *Peperomia pellucida* (L.) Kunth | Shiny bush (Eng.), Pansit-pansitan (Tag., Ilk.) | N | NL | H | L, S | High Uric Acid | 5 | Boil leaves or stem and drink decoction | 36 | 0.14 | 0.08 | 0.28 |
|  |  |  |  |  |  | L, S | Arthritis | 15 | Boil leaves or stem and drink decoction; Crush leaves or stem to extract the juice and apply to the affected area |  |  |  |  |
|  |  |  |  |  |  | L, S | Gout | 15 | Boil leaves or stem and drink decoction; Crush leaves or stem to extract the juice and apply to the affected area |  |  |  |  |
|  |  |  |  |  |  | L, S | Rheumatism | 15 | Boil leaves or stem and drink decoction; Crush leaves or stem to extract the juice and apply to the affected area |  |  |  |  |
|  |  |  |  |  |  | L, S | Kidney Stones | 16 | Boil leaves or stem and drink decoction |  |  |  |  |
|  |  |  |  |  |  | L, S | UTI | 16 | Boil leaves or stem and drink decoction |  |  |  |  |
|  |  |  |  |  |  | L, S | Fever | 21 | Boil leaves or stem and drink decoction |  |  |  |  |
| Piperaceae | *Piper betle* L. | Betel pepper (Eng.), Ikmo (Tag.), Gawed (Ilk.) | C | NL | V | L | Sore throat | 12 | Boil leaves and gargle with the decoction; Chew raw leaves | 11 | 0.04 | 0.02 | 0.24 |
|  |  |  |  |  |  | L | Gastritis | 13 | Boil and drink decoction |  |  |  |  |
|  |  |  |  |  |  | L | Diarrhea | 13 | Boil and drink decoction |  |  |  |  |
|  |  |  |  |  |  | L | Gas pain | 13 | Boil and drink decoction |  |  |  |  |
|  |  |  |  |  |  | L | Stomachache | 13 | Boil and drink decoction |  |  |  |  |
|  |  |  |  |  |  | L | Headache | 21 | Heat leaf on a hot surface and apply to the forehead |  |  |  |  |
|  |  |  |  |  |  | L | Bruises | 22 | Boil leaves and wash bruises with the decoction; Pound leaves into a paste and apply as poultice |  |  |  |  |
|  |  |  |  |  |  | L | Wounds | 22 | Boil leaves and wash wounds with the decoction; Pound leaves into a paste and apply as poultice |  |  |  |  |
| Plantaginaceae | *Scoparia dulcis* L. | Licorice weed (Eng.), Mala-anis (Tag.), Isisa (Ilk.) | Nu | NL | H | L | Toothache | 13 | Pound leaves into a paste and apply as poultice | 2 | 0.01 | 0.01 | 0.06 |
| Poaceae | *Bambusa spinosa*Roxb. ex Buch.-Ham. | Spiny bamboo (Eng.) Kawayan (Tag., Ilk.) | CNu | NL | T | L, S | Diarrhea | 13 | Boil leaves and drink decoction | 2 | 0.01 | 0.00 | 0.11 |
|  |  |  |  |  |  | L, S | Irregular menstruation | 16 | Boil leaves and drink decoction |  |  |  |  |
| Poaceae | *Chrysopogon aciculatus* (Retz.) Trin. | Love grass (Eng.), Amorseco (Tag.), Tinloy (Ilk.) | N | NL | Herb | L, R | Asthma | 12 | Boil leaves or roots and drink decoction | 11 | 0.04 | 0.04 | 0.19 |
|  |  |  |  |  |  | L, R | Gall bladder stone | 13 | Boil leaves or roots and drink decoction |  |  |  |  |
|  |  |  |  |  |  | L, R | Liver problem | 13 | Boil leaves or roots and drink decoction |  |  |  |  |
|  |  |  |  |  |  | L, R | UTI | 16 | Boil leaves or roots and drink decoction |  |  |  |  |
| Poaceae | *Cymbopogon citratus* (DC.) Stapf | Lemon grass (Eng.), Tanglad (Tag.), Baraniw (Ilk.) | CNu | NL | Herb | L | Flu | 1 | Boil leaves and use decoction for a warm or steam bath (*suob*). Can be used together with *V. negundo*, *B. balsamifera*, and *P. guajava* | 77 | 0.31 | 0.15 | 0.55 |
|  |  |  |  |  |  | L | Hypertension | 11 | Boil leaves and drink decoction |  |  |  |  |
|  |  |  |  |  |  | L | Colds | 12 | Boil leaves and drink decoction |  |  |  |  |
|  |  |  |  |  |  | L | Cough | 12 | Boil leaves and drink decoction |  |  |  |  |
|  |  |  |  |  |  | L | Sore throat | 12 | Boil leaves and drink decoction |  |  |  |  |
|  |  |  |  |  |  | L | Diarrhea | 13 | Boil leaves and drink decoction |  |  |  |  |
|  |  |  |  |  |  | L | Indigestion | 13 | Boil leaves and drink decoction |  |  |  |  |
|  |  |  |  |  |  | L | Stomachache | 13 | Boil leaves and drink decoction |  |  |  |  |
|  |  |  |  |  |  | L | UTI | 16 | Boil leaves and drink decoction |  |  |  |  |
|  |  |  |  |  |  | L | Post-partum Care | 18 | Boil leaves and use decoction for a warm or steam bath (*suob*). Can be used together with *V. negundo*, *B. balsamifera*, and *P. guajava* |  |  |  |  |
|  |  |  |  |  |  | L | Abdominal pain | 21 | Boil leaves and drink decoction |  |  |  |  |
|  |  |  |  |  |  | L | Fever | 21 | Boil leaves in water to make a decoction. Use water for bathing or steam bath (SUOB). Can be used together with *V. negundo*, *B. balsamifera*, and *P. guajava*. |  |  |  |  |
|  |  |  |  |  |  | L | Detoxification/ Cleansing | 00 | Boil leaves and drink decoction |  |  |  |  |
| Poaceae | *Echinochloa colonum* (L.) Link | Jungle rice (Eng.), Pulang puwit (Tag.), Dakayang (Ilk.) | N | LC | Herb | L, Se | Hypertension | 11 | Boil leaves or seeds and drink decoction | 3 | 0.01 | 0.01 | 0.12 |
|  |  |  |  |  |  | L, R | Arthritis | 15 | Boil leaves or roots and drink decoction |  |  |  |  |
| Poaceae | *Eleusine indica* (L.) Gaertn. | Wire grass (Eng.) Paragis (Tag., Ilk.) | Nu | LC | Herb | L | Diabetes | 5 | Boil leaves and drink decoction | 3 | 0.01 | 0.00 | 0.17 |
|  |  |  |  |  |  | L | Hypertension | 11 | Boil leaves and drink decoction |  |  |  |  |
|  |  |  |  |  |  | L | Rheumatism | 15 | Boil leaves and drink decoction |  |  |  |  |
| Poaceae | *Oryza sativa* L. | Rice (Eng.), Palay (Tag.), Pagay (Ilk.) | CNu | NL | Herb | Fr | Mumps | 1 | Mash boiled rice and apply directly to mumps | 18 | 0.07 | 0.06 | 0.32 |
|  |  |  |  |  |  | Fr | Anemia | 3 | Wash rice, collect starchy water, boil, cool and drink rice water |  |  |  |  |
|  |  |  |  |  |  | Fr | Blindness | 9 | Wash rice, collect starchy water, boil, cool, and use rice water to wash eyes |  |  |  |  |
|  |  |  |  |  |  | Fr | Skin Sores | 14 | Wash rice, collect starchy water, boil, cool, and use rice water to wash skin sores |  |  |  |  |
|  |  |  |  |  |  | Fr | Kidney Problems | 16 | Wash rice, collect starchy water, boil, cool and drink rice water |  |  |  |  |
| Poaceae | *Zea mays* L. | Corn (Eng.), Mais (Tag., Ilk.) | CNu | LC | Herb | Fr | Hypertension | 11 | Boil corn silk and drink decoction; Eat boiled corn kernels | 16 | 0.06 | 0.05 | 0.14 |
|  |  |  |  |  |  | Fr | Diuretic | 11 | Boil corn silk and drink decoction |  |  |  |  |
|  |  |  |  |  |  | Fr | Kidney Problem | 16 | Boil corn silk and drink decoction |  |  |  |  |
|  |  |  |  |  |  | Fr | UTI | 16 | Boil corn silk and drink decoction |  |  |  |  |
| Portulacaceae | *Portulaca oleracea* L. | Purselane weed (Eng.), Ulasiman (Tag.), Tabtabukol (Ilk.) | C | LC | H | L | Malaria | 1 | Boil leaves and drink decoction | 6 | 0.02 | 0.02 | 0.18 |
|  |  |  |  |  |  | S | Kidney problems | 16 | Boil stem and drink decoction |  |  |  |  |
|  |  |  |  |  |  | L | Bruises | 22 | Boil leaves and wash bruises with the decoction; Pound leaves into a paste and apply as poultice |  |  |  |  |
|  |  |  |  |  |  | L | Cuts | 22 | Boil leaves and wash cuts with the decoction; Pound leaves into a paste and apply as poultice |  |  |  |  |
| Rhamnaceae | *Ziziphus mauritiana* Lam. | Jujube plum (Eng.), Mansanitas (Tag., Ilk.) | Nu | LC | T | L | Diabetes | 5 | Boil leaves and drink decoction | 7 | 0.03 | 0.02 | 0.18 |
|  |  |  |  |  |  | L, Ba | Diarrhea | 13 | Boil leaves or bark and drink decoction |  |  |  |  |
|  |  |  |  |  |  | L, Fr | Fever | 21 | Boil leaves and drink decoction; Eat ripe fruit |  |  |  |  |
| Rubiaceae | *Ixora coccinea* L. | Ixora (Eng.), Santan (Tag., Ilk.) | CNu | NL | S | R | Dysentery | 1 | Boil flowers and roots and drink decoction. | 5 | 0.02 | 0.01 | 0.12 |
|  |  |  |  |  |  | L, F | Boils | 1 | Pound leaves and flowers into a paste and apply as poultice |  |  |  |  |
|  |  |  |  |  |  | F, R | Diarrhea | 13 | Boil flowers and roots and drink decoction. |  |  |  |  |
| Rubiaceae | *Morinda citrifolia* L. | Noni (Eng.), Tumbong-aso (Tag.), Apatot (Ilk.) | N | LC | S | Fr | Cancer | 2 | Extract juice from fruit and drink 1 glass/cup 1-2 times a day. | 7 | 0.03 | 0.02 | 0.24 |
|  |  |  |  |  |  | Fr | Diabetes | 5 | Extract juice from fruit and drink 1 glass/cup 1-2 times a day. |  |  |  |  |
|  |  |  |  |  |  | Fr | Hypertension | 11 | Extract juice from fruit and drink 1 glass/cup 1-2 times a day. |  |  |  |  |
|  |  |  |  |  |  | L, Fr | Arthritis | 15 | Extract juice from fruit and drink 1 glass/cup 1-2 times a day; Crush leaves and fruit to make a paste. Apply paste directly to affected part. |  |  |  |  |
|  |  |  |  |  |  | L, Fr | Gout | 15 | Extract juice from fruit and drink 1 glass/cup 1-2 times a day; Crush leaves and fruit to make a paste. Apply paste directly to affected part. |  |  |  |  |
| Rutaceae | *Citrus* × *aurantiifolia* (Christm.) Swingle | Lime (Eng.), Dayap (Tag.), Dalayap (Ilk.) | Nu | NL | S | Fr | Colds | 12 | Extract fruit juice and drink | 7 | 0.03 | 0.01 | 0.12 |
|  |  |  |  |  |  | Fr | Cough | 12 | Extract fruit juice and drink |  |  |  |  |
|  |  |  |  |  |  | Fr | Dizziness | 21 | Extract oil from the fruit rind and inhale the scent |  |  |  |  |
| Rutaceae | *Citrus* × *microcarpa* Bunge | Philippine Lime (Eng.), Kalamondin (Tag.), Kalamansi (Tag., Ilk.) | Nu | NL | S | Fr | Colds | 12 | Extract fruit juice, add honey and drink | 5 | 0.02 | 0.01 | 0.06 |
|  |  |  |  |  |  | Fr | Cough | 12 | Extract fruit juice, add honey and drink |  |  |  |  |
| Rutaceae | *Citrus maxima* (Burm.) Merr. | Pomelo (Eng.), Suha (Tag.), Lukban (Ilk.) | Nu | LC | T | Fr | Diabetes | 5 | Infuse leaves in hot water and drink infusion; Eat ripe fruit | 5 | 0.02 | 0.01 | 0.12 |
|  |  |  |  |  |  | L, Fr | Colds | 12 | Boil leaves and drink decoction |  |  |  |  |
|  |  |  |  |  |  | L, Fr | Cough | 12 | Boil leaves or bark and drink decoction |  |  |  |  |
| Sapotaceae | *Chrysophyllum cainito* L. | Star Apple (Eng.), Kaimito (Tag., Ilk.) | CNu | LC | T | L | Anemia | 3 | Boil leaves and drink decoction. Infuse leaves in water and drink infusion. Eat fruit. | 7 | 0.03 | 0.01 | 0.18 |
|  |  |  |  |  |  | L | Diabetes | 5 | Boil leaves and drink decoction |  |  |  |  |
|  |  |  |  |  |  | L, Ba | Diarrhea | 13 | Boil leaves or bark and drink decoction |  |  |  |  |
| Sapotaceae | *Pouteria campechiana*  (Kunth) Baehni | Egg Fruit Tree (Eng.), Tiesa (Tag., Ilk.) | CNu | LC | T | L | Diarrhea | 13 | Boil leaves and drink decoction | 1 | 0.00 | 0.00 | 0.06 |
| Solanaceae | *Nicotiana tabacum* L. | Tobacco (Eng.), Tabako (Tag., Ilk.) | Nu | NL | H | L | Boils | 1 | Pound leaves into a paste and apply as poultice | 7 | 0.03 | 0.02 | 0.12 |
|  |  |  |  |  |  | L | Weak teeth | 13 | Chew with *A. catechu*, *P. betel,* and lime |  |  |  |  |
| Solanaceae | *Solanum lycopersicum* L. | Tomato (Eng.), Kamatis (Tag., Ilk.) | CNu | NL | H | Fr | Burns | 22 | Pound fruit into a paste and apply as poultice | 5 | 0.02 | 0.01 | 0.06 |
|  |  |  |  |  |  | Fr | Cuts | 22 | Pound fruit into a paste and apply as poultice |  |  |  |  |
|  |  |  |  |  |  | Fr | Wounds | 22 | Pound fruit into a paste and apply as poultice |  |  |  |  |
| Solanaceae | *Solanum nigrum* L. | Black nightshade (Eng.), Lubi-lubi (Tag.), Am-amsi (Ilk.) | Nu | NL | H | L | Boils | 1 | Pound leaves into a paste and apply as poultice | 5 | 0.02 | 0.01 | 0.23 |
|  |  |  |  |  |  | L | Skin sores | 14 | Boil leaves and wash skin sores with the decoction; Pound leaves into a paste and apply as poultice |  |  |  |  |
|  |  |  |  |  |  | L | Snake bite | 22 | Boil leaves and wash snake bites with the decoction; Pound leaves into a paste and apply as poultice |  |  |  |  |
|  |  |  |  |  |  | L | *Pasma* | 00 | Boil leaves and use decoction for a warm bath |  |  |  |  |
| Verbenaceae | *Lantana camara* L. | Lantana (Eng.), Kantutay (Tag.), Bangbangsit (Ilk.) | Nu | NL | S | L | Fever | 21 | Boil leaves and drink decoction | 5 | 0.02 | 0.01 | 0.12 |
|  |  |  |  |  |  | L | Sprain | 22 | Pound leaves into a paste and apply as poultice |  |  |  |  |
|  |  |  |  |  |  | L | Wounds | 22 | Pound leaves into a paste and apply as poultice |  |  |  |  |
| Zingiberaceae | *Curcuma longa* L. | Turmeric (Eng.), Luyang dilaw (Tag.), Kulyaw (Ilk.) | Nu | DD | Herb | Rh | Flu | 1 | Boil rhizomes and drink or use decoction for a steam bath (*suob*) | 12 | 0.05 | 0.02 | 0.35 |
|  |  |  |  |  |  | Rh | Diabetes | 5 | Boil rhizomes and drink decoction |  |  |  |  |
|  |  |  |  |  |  | Rh | Hypertension | 11 | Boil rhizomes and drink decoction |  |  |  |  |
|  |  |  |  |  |  | Rh | Cough | 12 | Boil rhizomes and drink or use decoction for a steam bath (*suob*) |  |  |  |  |
|  |  |  |  |  |  | Rh | Sore throat | 12 | Boil rhizomes and drink or use decoction for a steam bath (*suob*) |  |  |  |  |
|  |  |  |  |  |  | Rh | Fever | 21 | Boil rhizomes and drink or use decoction for a steam bath (*suob*) |  |  |  |  |
|  |  |  |  |  |  | Rh | Bruises | 22 | Boil rhizomes and wash bruises with the decoction; Pound rhizomes into a paste and apply as poultice |  |  |  |  |
|  |  |  |  |  |  | Rh | Wounds | 22 | Boil rhizomes and wash wounds with the decoction; Pound rhizomes into a paste and apply as poultice |  |  |  |  |
| Zingiberaceae | *Zingiber officinale* Roscoe | Ginger (Eng.), Luya (Tag.), Laya (Ilk.) | Nu | DD | H | Rh | Hoarse voice | 12 | Boil rhizomes and drink decoction | 20 | 0.08 | 0.03 | 0.24 |
|  |  |  |  |  |  | Rh | Cough | 12 | Crush rhizomes to extract the juice and drink |  |  |  |  |
|  |  |  |  |  |  | Rh | Sore throat | 12 | Boil rhizomes and drink decoction; Crush rhizomes to extract the juice and drink |  |  |  |  |
|  |  |  |  |  |  | Rh | Indigestion | 13 | Boil rhizomes and drink decoction |  |  |  |  |
|  |  |  |  |  |  | Rh | Gas pain | 13 | Boil rhizomes and drink decoction |  |  |  |  |
|  |  |  |  |  |  | Rh | Rheumatism | 15 | Boil rhizomes and drink decoction; Crush rhizomes to extract the juice and drink |  |  |  |  |
|  |  |  |  |  |  | Rh | Bruises | 22 | Pound rhizomes into a paste and apply as poultice |  |  |  |  |

*LEGENDS:

†Origin – Native (N); Endemic (E); Naturalized (Nu); Cryptogenic (C); Cultivated, not Native (CN); Cultivated not Naturalized (CNu)

⸸IUCN – Endangered (EN); Least Concern (LC); Data Deficient (DD); Not Listed (NL)

‡Growth Forms – Herb (H); Tree (T); Shrub (S); Vine (V)

⁑Plant Parts – Leaf (L); Stem (S); Flower (F); Root (R); Fruit (Fr); Seed (Se); Bark (Ba); Bulb (Bu); Rhizome (Rh)

*DC – Disease category
