# Supplementary material for: Ethnobotanical survey and quantitative assessment of medicinal plants in landlocked communities of San Fernando, La Union, Philippines
Source: Front Pharmacol. 2025 Nov 13;16:1670496. doi: 10.3389/fphar.2025.1670496 (PMC12657414; doi:10.3389/fphar.2025.1670496)
Supplement: Supplementary file 5 [file Table4.docx]

Supplementary Material 4. Disease Categories, disease or purpose associated with each category, number of species used per disease category, UR, ICF, species with the highest ICF and its purpose, FL%, species with the highest FL% and its purpose.

| No. | Disease Category | †Disease/Purpose | Number of species used | UR | ICF | Species with the highest citation | Purpose | FL (%) | Species with the highest FL | Purpose |
| --- | --- | --- | --- | --- | --- | --- | --- | --- | --- | --- |
| 1 | Certain Infectious or Parasitic Diseases | Abscess (1), Amoebiasis (1), Boils (30), Dengue fever (28), Dysentery (18), Flu (162), Intestinal worms (2), Malaria (4), Mumps (10), Relapsing fever (3), Ringworm (1), Scabies (2), Warts (1) | 35 | 387 | 0.87 | *Vitex arvensis* Gentallan, Sengun & M.B. Bartolome  (89) | Flu | 71.43 | *Hibiscus rosa-sinensis* L. | Boils |
| 2 | Neoplasms | Cancer (5) | 2 | 81 | 0.75 | *Annona muricata* L. (4) | Cancer | 14.29 | *Morinda citrifolia* L. | Cancer |
| 3 | Diseases of the Blood and Blood-Forming Organs | Anemia (7) | 3 | 18 | 0.67 | *Oryza sativa* L. (3) | Anemia | 28.57 | *Chrysophyllum cainito* L. | Anemia |
| 4 | Diseases of the Immune System | Allergies (2) | 2 | 10 | 1.00 | *Ocimum tenuiflorum* L. (2) | Allergies | 20.00 | *Ocimum tenuiflorum* L. | Allergies |
| 5 | Endocrine, Nutritional, or Metabolic Diseases | Beri-beri (2), Diabetes (123), High Cholesterol (21), High Uric Acid (3) | 26 | 75 | 0.83 | *Andrographis paniculata* (Burm.f.) Wall. ex Nees (43) | Diabetes | 66.67 | *Colocasia esculenta* (L.) Schott | Diabetes |
| 9 | Diseases of the Visual System | Eye Infection (3), Sore eyes (1), Blindness (1), Puffy eyes (3) | 3 | 12 | 0.57 | *Calophyllum inophyllum* L. (3) | Eye Infection | 25.00 | *Calophyllum inophyllum* L. | Eye Infection |
| 11 | Diseases of the Circulatory System | Diuretic (7), Edema (1), Hypertension (116), Varicose veins (1) | 26 | 75 | 0.80 | *Andrographis paniculata* (Burm.f.) Wall. ex Nees (31) | Hypertension | 50.00 | *Malvastrum coromandelianum* (L.) Garcke | Hypertension |
| 12 | Diseases of the Respiratory System | Asthma (42), Bronchitis (4), Colds (15), Cough (352), Hoarse voice (1), Sore throat (1), Tonsilitis (5) | 26 | 387 | 0.95 | *Vitex arvensis* Gentallan, Sengun & M.B. Bartolome (157) | Cough | 100.00 | *Premna odorata* Blanco | Cough |
| 13 | Diseases of the Digestive System | Constipation (15), Diarrhea (68), Gall bladder stone (3), Gas pain (8), Gastritis (1), Hyperacidity (2), Indigestion (8), Irregular bowel movement (2), Liver problem (2), Stomachache (18), Toothache (5), Vomiting (2), Weak teeth (7) | 42 | 83 | 0.71 | *Psidium guajava* L. (14) | Diarrhea | 100.00 | *Cassia fistula* L. | Constipation |
|  |  |  |  |  |  |  |  | 100.00 | *Scoparia dulcis* L. | Toothache |
|  |  |  |  |  |  |  |  | 100.00 | *Pouteria campechiana*  (Kunth) Baehni | Diarrhea |
| 14 | Diseases of the Skin | Dandruff (6), Dry hair (3), Dry skin (7), Eczema (2), Hair fall (1), Hives (7), Rashes (1), Skin diseases (1), Skin infection (1), Skin sores (4), Skin ulcer (3) | 14 | 29 | 0.76 | *Pandanus amaryllifolius* Roxb. ex Lindl.  (5) | Dandruff | 42.86 | *Phyllanthus acidus* (L.) Skeels | Hives |
| 15 | Diseases of the Musculoskeletal System or Connective Tissue | Arthritis (20), Gout (9), Inflamed/Swollen Muscles (2), Muscle cramps (2), Muscle Pain (1), Rheumatism (21) | 14 | 36 | 0.91 | *Peperomia pellucida* (L.) Kunth (14) | Arthritis | 66.67 | *Echinochloa colonum* (L.) Link | Arthritis |
| 16 | Diseases of the Genitourinary System | Difficulty in Urinating (20), Dysmenorrhea (5), Irregular menstruation (12), Kidney Problem (40), Kidney Stones (35), UTI (63) | 17 | 223 | 0.79 | *Blumea balsamifera* (L.) DC. (28) | Kidney Stones | 50.00 | *Artemisia indica* Willd. | Irregular menstruation |
|  |  |  |  |  |  |  |  | 50.00 | *Bambusa spinosa* Roxb. ex Buch.-Ham. | Irregular menstruation |
|  |  |  |  |  |  |  |  | 50.00 | *Malvastrum coromandelianum* (L.) Garcke | UTI |
| 18 | Pregnancy, Childbirth, or the Puerperium | Post partum care (10), Increase breastmilk production (10) | 5 | 21 | 0.81 | *Moringa oleifera*Lam. (10) | Increase Breastmilk Production | 47.62 | *Moringa oleifera* Lam. | Increase Breastmilk Production |
| 19 | Certain Conditions Originating in the Perinatal Period | Abortifacient (2) | 1 | 9 | 0.55 | *Swietenia mahagoni* (L.) Jacq. (2) | Abortifacient | 22.22 | *Swietenia mahagoni* (L.) Jacq. | Abortifacient |
| 21 | Symptoms, Signs, or Clinical Findings Not Elsewhere Classified | Abdominal pain (5), Dizziness (3), Fainting (1), Fever (227), Headache (20), Puffy eyes (1), Skin ulcer (1) | 35 | 387 | 0.87 | *Vitex arvensis* Gentallan, Sengun & M.B. Bartolome (105) | Fever | 100.00 | *Cordia dichotoma* G.Forst. | Headache |
| 22 | Injury, Poisoning, and Certain Other Consequences of External Causes | Bruises (28), Bumps (29), Burns (19), Cuts (5), Insect bite (3), Scalds (3), Snake bite (3), Sprain (5), Sunburn (4), Wound (78) | 35 | 47 | 0.81 | *Allium tuberosum* Rottler ex Spreng. (29) | Bumps | 100.00 | *Tabernaemontana pandacaqui* Poir. | Wounds |
|  |  |  |  |  |  |  |  | 100.00 | *Chromolaena odorata* (L.) R.M.King & H.Rob. | Wounds |
|  |  |  |  |  |  |  |  | 100.00 | *Ficus septica*  Burn. f. | Wound |
|  |  |  |  |  |  |  |  | 100.00 | *Passiflora foetida* L. | Wounds |
| 0 | Other Diseases | Detoxification/Cleansing (1), *Nakablaawan* (2), *Pasma* (9) | 6 | 387 | 0.55 | *Vitex arvensis*Gentallan, Sengun & M.B. Bartolome (5) | *Pasma* | 20.00 | *Solanum nigrum* L. | *Pasma* |

*LEGEND:

†Disease/Purpose – the number of times the disease was mentioned is indicated in parentheses.
